# Reduction of the Diazo Functionality of α-Diazocarbonyl Compounds into a Methylene Group by NH_3_BH_3_ or NaBH_4_ Catalyzed by Au Nanoparticles

**DOI:** 10.3390/nano11010248

**Published:** 2021-01-18

**Authors:** Marios Kidonakis, Manolis Stratakis

**Affiliations:** Department of Chemistry, University of Crete, 71003 Voutes, Heraklion, Greece; makidonakis@uoc.gr

**Keywords:** α-diazocarbonyl compounds, transfer hydrogenation, boron hydrides, heterogeneous catalysis, supported Au nanoparticles

## Abstract

Supported Au nanoparticles on TiO_2_ (1 mol%) are capable of catalyzing the reduction of the carbene-like diazo functionality of α-diazocarbonyl compounds into a methylene group [C=(N_2_) → CH_2_] by NH_3_BH_3_ or NaBH_4_ in methanol as solvent. The Au-catalyzed reduction that occurs within a few minutes at room temperature formally requires one hydride equivalent (B-H) and one proton that originates from the protic solvent. This pathway is in contrast to the Pt/CeO_2_-catalyzed reaction of α-diazocarbonyl compounds with NH_3_BH_3_ in methanol, which leads to the corresponding hydrazones instead. Under our stoichiometric Au-catalyzed reaction conditions, the ketone-type carbonyls remain intact, which is in contrast to the uncatalyzed conditions where they are selectively reduced by the boron hydride reagent. It is proposed that the transformation occurs via the formation of chemisorbed carbenes on Au nanoparticles, having proximally activated the boron hydride reagent. This protocol is the first general example of catalytic transfer hydrogenation of the carbene-like α -ketodiazo functionality.

## 1. Introduction

The reduction of the carbene-like diazo functionality into a methylene group [C=(N_2_) → CH_2_] in α-diazocarbonyl compounds is not a well-studied transformation in the past, and there are scattered examples in the literature. Decades ago, the Wolfrom method [1] was reported, which employs hydriodic acid as the reducing agent and then a protocol involving catalytic amounts of diethyl peroxydicarbonate in isopropanol [2]. The interesting transformation of α-diazo-β-hydroxy esters into β-keto esters [3,4] catalyzed by Rh_2_(OAc)_4_ could be seen as a relevant example and apparently involves an intramolecular transfer hydrogenation. Bu_3_SnH has been also used as reducing agent with Cu(acac)_2_ as catalyst under irradiation [5], as well as a series of early transition metallocenes in the presence of H_2_O [6]. Finally, the relevant to this concept Pd/C-catalyzed hydrogenation of a specific type of diazo compounds (α-diazo-β-hydroxy esters) [7] appears as a practical reductive method providing β-hydroxy esters in moderate to good yields.

**Scheme 1:**
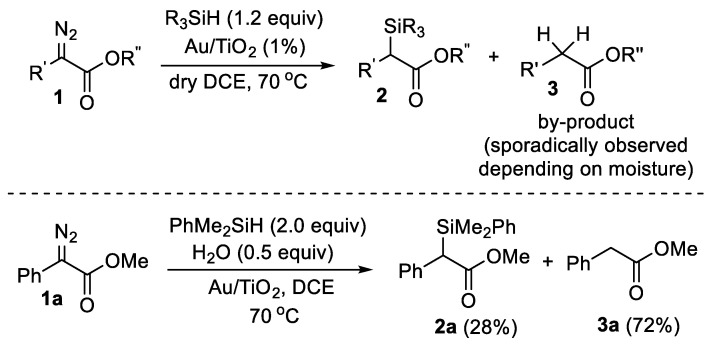
Hydrosilylation of α-diazo carbonyl compounds catalyzed by Au/TiO_2_; reversal of chemoselectivity of the attempted hydrosilylation of diazo compound **1a** in the presence of 0.5 equivalents of H_2_O.

Recently, we presented the first example of Au-catalyzed hydrosilylation of α-diazocarbonyl compounds using recyclable and reusable Au nanoparticles on TiO_2_ as the active catalytic sites (Scheme 1) [8]. This reaction proceeds in short reaction times in anhydrous 1,2-dichloroethane (DCE) affording α-silyl carbonyl compounds in high yields. It is most likely that this transformation involves not only the activation of the diazo compounds on Au NPs forming chemisorbed Au-carbenes [9] but proximally chemisorbed hydrosilanes as well. The only side product of the hydrosilylation reaction is the replacement of the diazo group by two hydrogen atoms [C=(N_2_) → CH_2_]. This side pathway depends on the amount of moisture in the solvent and apparently arises from the chemisorbed H species on the Au nanoparticle. This observation initiated our efforts to develop a new methodology for the reduction of the diazo functionality of α-diazocarbonyl compounds into a methylene group. When we contacted the Au/TiO_2_-catalyzed hydrosilylation of diazo compound **1a** in DCE in the additional presence of H_2_O (0.5 equivalent), the product selectivity altered, affording the reduced ester **3a** in 72% relative yield versus 28% of the anticipated hydrosilylation product **2a**. Although the relative yield of **3a** can be further increased by increasing the equivalents of water added into the reaction mixture, at the same time, a high excess of hydrosilane should be used, as hydrosilanes react with H_2_O (a reaction that is also catalyzed by Au/TiO_2_). Thus, we sought for an alternative and cheaper reducing agent to replace hydrosilane and establish a more practical methodology to achieve this reductive transformation.

Over the past two decades, supported Au nanoparticles (Au NPs) and other nano Au(0) materials have attracted significant attention due to their surprising ability to catalyze a plethora of organic transformations, despite the metallic character of Au atoms on nanoparticles [10,11,12,13], including transfer hydrogenation reductive processes [14,15]. Given the heterogeneous nature of Au NP-catalyzed processes and that they can be easily recycled and reused on the vast majority of the reported protocols, they can be considered as benign green catalysts.

## 2. Results

**Scheme 2:**
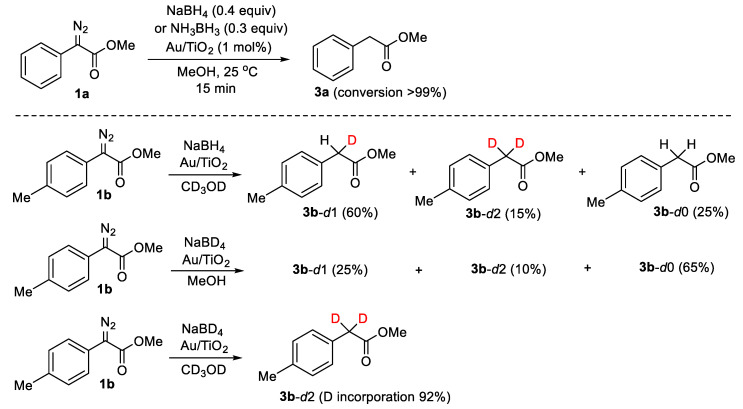
Reduction of α-diazocarbonyl compound **1a** by boron hydride reagents in methanol catalyzed by Au/TiO_2_, and D-labeling experiments in the reduction of compound **1b**.

At the outset, we investigated the possible Au/TiO_2_-catalyzed reduction of the model compound **1a** into the desired product **3a**, using the readily available boron hydride reagents NH_3_BH_3_ and NaBH_4_. Ammonia borane complex [16,17] as well as sodium borohydride [18] have been used efficiently in the past for the reduction of alkynes or nitro compounds using Au/TiO_2_ as catalyst. While no reaction occurs in a series of dry aprotic solvents (dichloromethane, diethyl ether, hexane, or benzene), to our pleasure, we found that the treatment of **1a** with NH_3_BH_3_ (0.4 molar equivalent) or NaBH_4_ (0.3 molar equivalent) in MeOH for 15 min at room temperature resulted in the quantitative formation of the reduced product **3a** (Scheme 2). In the absence of Au/TiO_2_ or in the presence of the support itself (TiO_2_, routile, or anatase), no transformation took place even after prolonged reaction times (12 h) at 50 °C, which is indicative that Au NPs are the catalytic sites.

The amounts of boron hydride reagents that are necessary to achieve a quantitative reduction indicate that one hydride equivalent from either NH_3_BH_3_ or NaBH_4_ is required; thus, the second hydrogen atom on the product arises from the protic solvent (methanol). To test this hypothesis, we performed the reduction of diazo compound **1b** with NaBH_4_ in methanol-*d*_4_ (Scheme 2). Analysis of the reaction mixture revealed the presence of D atoms in the products. The monodeuterated **3b**-*d*_1_ was formed in 60% relative yield, along with dideuterated **3b**-*d*_2_ (15%) and non-deuterated **3b**-*d*_0_ (25%). The incorporation of H atoms from protic solvents in the Au/TiO_2_-catalyzed semireduction of alkynes using NH_3_BH_3_ has been already shown by our group in the past [16]. An analogous H/D scrabbling pattern in products has been reported in the Pd-catalyzed hydrogenation of *N*-tosylhydrazones in MeOD [19]. However, a different product distribution was seen in the reduction of **1b** with NaBD_4_ (98% D) in methanol-*d*_0_ (Scheme 2). Under these conditions, the fully protonated product **3b**-*d*_0_ was the major one (65% relative yield), along with **3b**-*d*_1_ (25%) and **3b**-*d*_2_ (10%). Finally, the reduction of **1b** with NaBD_4_ in methanol-*d*_4_ afforded product **3a**-*d*_2_ with 92% deuterium incorporation. The incomplete deuteration in the latter experiment is attributed to traces of moisture present in the reaction medium.

These observations support our consideration regarding the origins of the two hydrogen atoms: one hydride from NaBH_4_ or NH_3_BH_3_ and one proton from MeOH. In the accompanying mechanistic analysis, we will comment on the differences in the product distribution between **3b**-*d*_2_, **3b**-*d*_1_, and **3b**-*d*_0_, upon replacing NaBH_4_ with NaBD_4_ or methanol-*d*_0_ with methanol-*d*_4_.

**Scheme 3:**
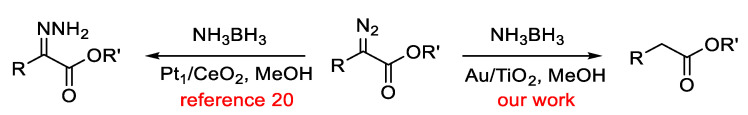
The reaction of α-diazocarbonyl compounds with NH_3_BH_3_ in methanol under different catalytic conditions.

The observed result is outstanding, given that the reduction is very fast, occurs at room temperature with low loading of the catalyst, and the amount of the reducing agent is stoichiometric in terms of hydride equivalents. Thus, for instance, one molar equivalent of NH_3_BH_3_ can formally achieve the reduction of three molar equivalents of the α-diazocarbonyl compound. The catalyst can be recycled at the end of each reaction by simple filtration and then reused after being washed with dichloromethane and dried in the oven at 90 °C for 2 h. We were able to perform five consecutive catalytic runs with the same batch of catalyst without observing any obvious deterioration in terms of yields. We emphasize herein the unique nature of our catalytic system, as it known [20] that in the analogous treatment of α-diazocarbonyl compounds with three molar equivalents of NH_3_BH_3_ under single-atom platinum on ceria catalysis conditions (Pt_1_/CeO_2_), the corresponding hydrazones are formed instead, as seen in Scheme 3.

**Figure 1:**
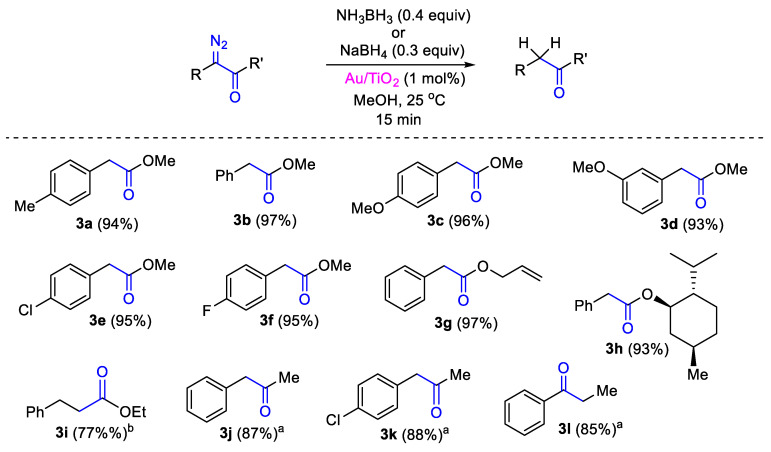
Reduction of α-diazocarbonyl compounds by NH_3_BH_3_ or NaBH_4_ catalyzed by Au/TiO_2_. ^a^ With one additional B-H equivalent, the corresponding alcohols of the ketones **3j**–**l** were isolated in >90% yield. ^b^ In this case, the corresponding hydrazone (Ε/Ζ = 85/15) was formed in 15% relative yield using NH_3_BH_3_ and in 25% with NaBH_4_. The isolated yield refers to the reaction with NH_3_BH_3_.

Subsequently, we examined the reduction of a series of α-diazocarbonyl compounds. The results are summarized in Figure 1. In all cases, the reaction proceeds smoothly within 15 min, the transformation is quantitative, and the isolated yields are very high, as the products do not essentially require any chromatographic purification in the majority of the cases. Noteworthily, the transformation is highly chemoselective given that the reduction of the generated Au-carbenes is faster than that of the ketone moiety. Thus, the reaction of α-diazo ketones **1j**–**l** with 0.35–0.4 molar equivalents of NH_3_BH_3_ or with 0.25–0.30 molar equivalents of NaBH_4_ solely provided ketones **3j**–**l**, respectively, leaving the ketone moiety intact. In the case of using excess of reducing agent (≈2.5 hydride equivalents), both the diazo and the ketone functionalities are reduced quantitatively to the corresponding alcohols. In the literature, it is well established that in the reduction of α-diazocarbonyl compounds with NaBH_4_, having an extra ketone carbonyl functionality, the diazo group remains intact [21]. The same holds in their reduction using biocatalytic conditions (ketoreductases, NADPH [22]). Notably, we found that in the absence of Au/TiO_2_, the reaction of **1l** with NH_3_BH_3_ or NaBH_4_ yields a complex mixture of products, and this is quite reasonable given the expected instability of the anticipated α-diazo alcohol. Additionally, while the results shown in Figure 1 were done at 0.2–0.3 mmol scale of the α-diazocarbonyl compounds, we performed the reduction of compound **1a** in a 3 mmol scale at 0 °C, producing the methylene product **3a** in almost quantitative yield within 15 min, which shows that our protocol is operational for larger-scale experiments.

**Scheme 4:**
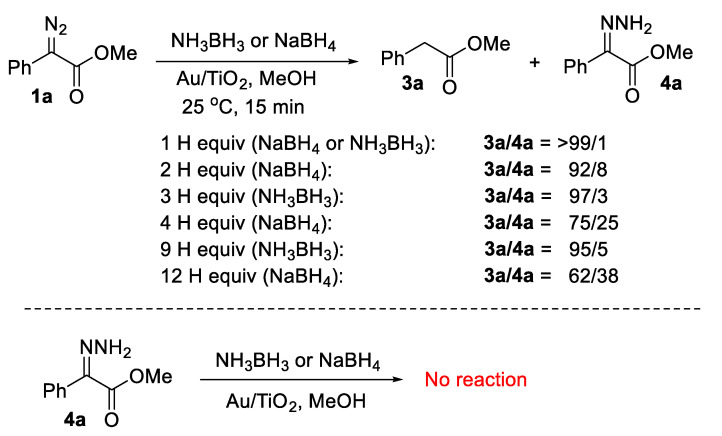
Au/TiO_2_-catalyzed reaction of **1a** with excess of NaBH_4_ or NH_3_BH_3_, and a proof that hydrazones are not reaction intermediates.

**Scheme 5:**
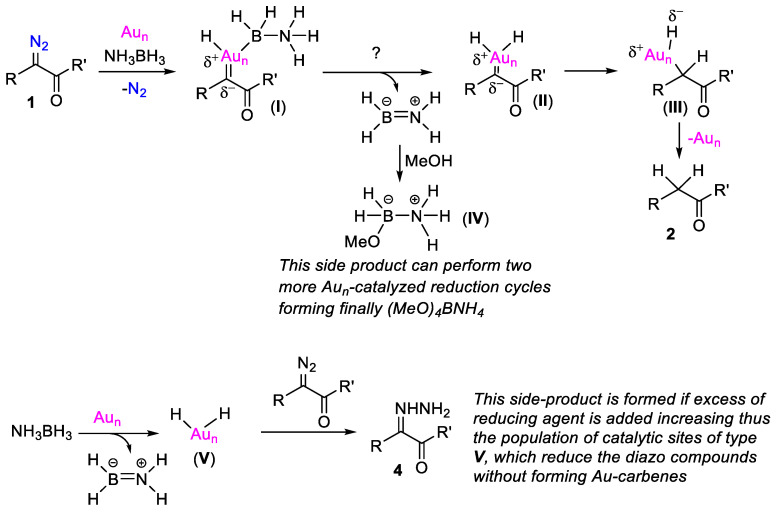
Mechanistic considerations in the reduction of α-diazocarbonyl compounds with NH_3_BH_3_ catalyzed by Au/TiO_2_ (the catalytic sites of nanoparticles are indicated as Au_n_).

As noted earlier in Scheme 3, hydrazones are formed exclusively in the Pt_1_/CeO_2_-catalyzed reaction of α-diazocarbonyl compounds using three molar equivalents of ammonia borane in MeOH [20]. We emphasize that in case of using an excess of reducing agent in our Au/TiO_2_-catalyzed transformation, the corresponding hydrazones were seen as minor products. For instance, in the reduction of substrate **1a**, we observed the following. Using 0.5–0.6 molar equivalents of NaBH_4_ (≈100% excess in terms of hydride equivalents), hydrazone **4a** [20] was observed in 8% yield relative to product **3a**. With 1.2 molar equivalents, **4a** is formed in 25% relative yield and with three molar equivalents in 38% (Scheme 4). On the other hand, using excess of NH_3_BH_3_, the reaction is more selective, with byproduct **4a** being formed in merely 3% relative yield if using 1.0 molar equivalent of ammonia borane and 5% relative yield with 3.0 molar equivalents. The only substrate that exhibited an obviously higher selectivity in terms of hydrazone formation was the α-diazocarbonyl compound **1i**. The treatment of **1i** with one hydride equivalent from NH_3_BH_3_ or NaBH_4_ resulted in the partial formation of the corresponding hydrazone **4i** [23] (*E*/*Z* = 85/15, see Supplementary Materials) in 15% and 25% relative yield, respectively. Hydrazone **4a** was isolated, and we found that it does not lead to product **3a** either upon treatment with 1 mol% Au/TiO_2_ under conditions identical to our protocol reaction or in the presence of the reductants (NH_3_BH_3_ or NaBH_4_) again in the presence of Au/TiO_2_ (MeOH, 15 min, 25 °C), and it is recovered intact (Scheme 4). Therefore, it is not a reaction intermediate. The same was observed with hydrazone **4i**. We comment on the origins of formation of the hydrazone side products in the accompanying mechanistic discussion and in Scheme 5.

The possible mechanistic scenario of the reductive process is presented in Scheme 5. Based on previous studies regarding the Au/TiO_2_-catalyzed reaction of α-diazocarbonyl compounds with hydrosilanes where Au-carbenes were invoked as intermediates [8], and the Au/TiO_2_-catalyzed semihydrogenation of alkynes with NH_3_BH_3_ [16] where Au-hydrides are the reactive species, we envision the formation of intermediate **I**, which can be described as a Schrock-type carbene [24] having chemisorbed on the electrophilic Au nanoparticle, which is a molecule of NH_3_BH_3_ on the same catalytic site. The elimination of NH_2_BH_2_ from **I** generates Au-dihydride intermediate **II**, which delivers the two H atoms on the former carbene carbon atom presumably in a stepwise manner (via intermediate **III**), thus leading to the final reduction product, with its concomitant liberation from the surface of nanoparticle Au_n_. The NH_2_BH_2_ reacts with methanol, providing the methoxy-bearing amine borane adduct **IV** shown in Scheme 5, which possesses even more reactive hydrides relative to NH_3_BH_3_. Amine borane **IV** reduces a second molecule of α-diazocarbonyl compound through a similar catalytic cycle on Au_n_, and it is transformed into NH_2_BH(OMe)_2_, which finally undergoes a third Au_n_-catalyzed reduction round. The final fate of ammonia borane is its transformation into NH_4_B(OMe)_4_ [25], which is proved by ^11^B NMR (see Supplementary Materials). Similarly, the final fate of NaBH_4_ is the formation of the analogous salt NaB(OMe)_4_, which resonates at about 3 ppm [26] in ^11^B NMR (see Supplementary Materials). If an excess of reducing agent is added, then the population of Au nanoparticle dihydro sites (intermediate **V**) increases, and subsequently, these sites can reduce the α-diazocarbonyl compounds through a side pathway that does not require their chemisorption on an Au nanoparticle. This pathway is similar to what was suggested in the Pt_1_/CeO_2_-catalyzed reduction of the α-diazocarbonyl compounds with NH_3_BH_3_ [20].

The D-labeling experiments presented in Scheme 2 can be rationalized, taking into account the proposed mechanism. Thus, in the reduction of **1b** with NaBH_4_ in methanol-*d*_4_, the major product is **3b**-*d*_1_ having one H from a B-H bond and one D from the O-D functionality of the solvent. The observation in parallel of the disproportionation products **3b**-*d*_2_ and **3b**-*d*_0_ implies that boron hydrides are partially exchanged by D atoms from the O-D functionality of the protic solvent under the reaction conditions; likewise, silicon-hydrides do in the Au/TiO_2_-catalyzed reduction of quinolines [27]. Notably, in analogous metal-catalyzed labeling experiments such as in the semihydrogenation of alkynes employing R_3_SiH(D) [28] or NaBH_4_(D_4_) [25], and MeOH(D) or H_2_(D_2_)O as the proton sources, no analogous isotopic scrabbling is observed. The H/D exchange of boron hydrides is very obvious in the reduction of **1b** with NaBD_4_ in CH_3_OH, where the fully protonated **3b**-*d*_0_ is the major product, while **3b**-*d*_0_ and **3b**-*d*_2_ are the minor ones. The differences in product distribution among these two labeling experiments are rather related to the different degree of Au/TiO_2_-catalyzed H/D exchange processes of boron-H(D) reagents from the solvent [MeOH or MeOH-*d*_4_], arising from intrinsic isotope effects.

## 3. Experimental Section

### 3.1. Catalyst

The supported Au nanoparticles on TiO_2_ having a gold content ≈1 wt%, which were used in our studies, are commercially available from Strem Chemicals Inc. (Newburyport, MA, USA) The average crystallite size of Au nanoparticles is ≈2–3 nm. Prior to use, they were grinded in a mortar, and the resulting purple dust was kept in a dark-colored bottle.

### 3.2. Reactants

The majority of α-diazocarbonyl compounds used in this study were available from previous studies in our lab [8]. The rest of them were prepared by α-diazotization of the corresponding carbonyl compounds with 4-acetamidobenzenesulfonyl azide in the presence of DBU as base [29]. Copies of the ^1^H and ^13^C NMR spectra of the α-diazocarbonyl compounds can be found in the Supplementary Materials.

### 3.3. Catalytic Reactions

Typical procedure of the title reaction: In a vial are placed 0.3 mmol of the α-diazocarbonyl compound, 0.09 mmol (3.5 mg) of NaBH_4_ (0.3 equiv) or 0.12 mmol (3.7 mg) of NH_3_BH_3_ (0.4 equiv), and 70 mg of Au/TiO_2_ (1.0 mol% in Au, as the catalytic system contains 1% Au *w*/*w*) in 0.5 mL of methanol. The heterogeneous mixture is stirred for 15 min at room temperature and within that period occurs complete consumption of the diazo compound (the supernatant reaction mixture turns to colorless). Then, the slurry is filtered with the aid of 2 mL of dichloromethane or any other volatile solvent such as diethyl ether under a low pressure through a short pad of silica gel, and the filtrate is evaporated. No chromatographic purification of the products is required in most of the cases, as the reactions are quantitative and yield only one product.

### 3.4. Characterization of Products

*Methyl 2-phenylacetate* (**3a**). ^1^H NMR (500 MHz, CDCl_3_): 7.35–7.27 (m, 5H), 3.70 (s, 3H), 3.64 (s, 2H); ^13^C NMR (125 MHz, CDCl_3_): 172.0, 133.9, 129.2, 128.6, 127.1, 52.0, 41.2.

*Methyl 2-(p-tolyl)acetate* (**3b**). ^1^H NMR (500 MHz, CDCl_3_): 7.18 (d, *J* = 8.0 Hz, 2H), 7.13 (d, *J* = 8.0 Hz, 2H), 3.68 (s, 3H), 3.59 (s, 2H), 3.33 (s, 3H); ^13^C NMR (125 MHz, CDCl_3_): 172.2, 136.7, 130.9, 129.2, 129.0, 51.9, 40.7, 21.0.

*Methyl 2-(4-methoxyphenyl)acetate* (**3c**). ^1^H NMR (500 MHz, CDCl_3_): 7.20 (d, *J* = 8.5 Hz, 2H), 6.86 (d, *J* = 8.5 Hz, 2H), 3.79 (s, 3H), 3.69 (s, 3H), 3.57 (s, 2H); ^13^C NMR (125 MHz, CDCl_3_): 172.3, 158.6, 130.2, 126.0, 113.9, 55.1, 51.9, 40.2.

*Methyl 2-(3-methoxyphenyl)acetate* (**3d**). ^1^H NMR (500 MHz, CDCl_3_): 7.24 (t, *J* = 8.0 Hz, 1H), 6.88–6.81 (m, 3H), 3.80 (s, 3H), 3.70 (s, 3H), 3.61 (s, 2H); ^13^C NMR (125 MHz, CDCl_3_): 171.8, 159.6, 135.3, 129.5, 121.5, 114.8, 112.5, 55.1, 52.0, 41.1.

*Methyl 2-(4-chlorophenyl)acetate* (**3e**). ^1^H NMR (500 MHz, CDCl_3_): 7.29 (d, *J* = 8.5 Hz, 2H), 7.21 (d, *J* = 8.5 Hz, 2H), 3.69 (s, 3H), 3.60 (s, 2H); ^13^C NMR (125 MHz, CDCl_3_): 171.6, 133.1, 132.3, 130.6, 128.7, 52.1, 40.4.

*Methyl 2-(4-fluorophenyl)acetate* (**3f**). ^1^H NMR (500 MHz, CDCl_3_): 7.26–7.23 (m, 2H), 7.01 (t, *J* = 8.5 Hz, 2H), 3.70 (s, 3H), 3.60 (s, 2H); ^13^C NMR (125 MHz, CDCl_3_): 171.9, 161.9 (d, *J*_C-F_ = 243.5 Hz), 130.8 (d, *J*_C-F_ = 8.0 Hz), 129.6 (d, *J*_C-F_ = 3.5 Hz), 115.4 (d, *J*_C-F_ = 21.5 Hz), 52.1, 40.2.

*Allyl 2-phenylacetate* (**3g**). ^1^H NMR (500 MHz, CDCl_3_): 7.35–7.28 (m, 5H), 5.95–5.87 (m, 1H), 5.28 (qd, *J*_1_ = 17.5 Hz, *J*_2_ = 1.5 Hz, 1H), 5.22 (qd, *J*_1_ = 10.5 Hz, *J*_2_ = 1.5 Hz, 1H), 4.61 (td, *J*_1_ = 6.0 Hz, *J*_2_ = 1.5 Hz, 2H), 3.66 (s, 2H); ^13^C NMR (125 MHz, CDCl_3_): 171.2, 133.9, 132.0, 129.2, 128.5, 127.1, 118.2, 65.4, 41.3.

*(1R,2S,5R)-2-Isopropyl-5-methylcyclohexyl 2-phenylacetate* (**3h**). ^1^H NMR (500 MHz, CDCl_3_): 7.34–7.24 (m, 5H), 4.71–4.66 (m, 1H), 3.61 (d, *J* = 16.0 Hz, 1H), 3.60 (d, *J* = 16.0 Hz, 1H), 1.99–1.96 (m, 1H), (1.77–1.71, m, 1H), 1.70–1.63 (m, 2H), 1.51–1.43 (m, 1H), 1.39–1.33 (m, 1H), 1.08–0.97 (m, 2H), 0.90 (d, *J* = 7.0 Hz, 3H), 0.84 (d, *J* = 7.0 Hz, 3H), 0.79 (d, *J* = 7.0 Hz, 3H); ^13^C NMR (125 MHz, CDCl_3_): 171.1, 134.3, 129.1, 128.4, 126.9, 74.6, 47.0, 41.8, 40.7, 34.2, 31.3, 26.1, 23.4, 22.0, 20.7, 16.2.

*Ethyl 3-phenylpropanoate* (**3i**). ^1^H NMR (500 MHz, CDCl_3_): 7.30–7.19 (m, 5H), 4.13 (q, *J* = 7.0 Hz, 2H), 2.96 (t, *J* = 7.5 Hz, 2H), 2.62 (t, *J* = 7.5 Hz, 2H), 1.24 (t, *J* = 7.0 Hz, 3H); ^13^C NMR (125 MHz, CDCl_3_): 172.9, 140.6, 128.5, 128.3, 126.2 60.4, 35.9, 31.0, 14.2.

*1-Phenylpropan-2-one* (**3j**). ^1^H NMR (500 MHz, CDCl_3_): 7.34 (t, *J* = 7.5 Hz, 2H), 7.28 (t, *J* = 7.5 Hz, 1H), 7.21 (d, *J* = 7.5 Hz, 2H), 3.70 (s, 2H), 2.16 (s, 3H); ^13^C NMR (125 MHz, CDCl_3_): 206.4, 134.2, 129.4, 128.8, 127.1, 51.0, 29.3.

*1-(4-Chlorophenyl)propan-2-one* (**3k**). ^1^H NMR (500 MHz, CDCl_3_): 7.30 (t, *J* = 8.5 Hz, 2H), 7.12 (t, *J* = 8.5 Hz, 2H), 3.67 (s, 2H), 2.16 (s, 3H); ^13^C NMR (125 MHz, CDCl_3_): 205.6, 133.0, 132.5, 130.7, 128.8, 50.0, 29.4.

*Propiophenone* (**3l**). ^1^H NMR (500 MHz, CDCl_3_): 7.97 (d, *J* = 8.5 Hz, 2H), 7.55 (t, *J* = 7.5 Hz, 1H), 7.46 (d, *J* = 7.5 Hz, 2H), 3.02 (q, *J* = 7.5 Hz, 2H), 1.24 (t, *J* = 7.5 Hz, 3H); ^13^C NMR (125 MHz, CDCl_3_): 200.8, 136.9, 132.9, 128.5, 128.0, 31.8, 8.2.

*Methyl (E)-2-hydrazono-2-phenylacetate* (**4a**). ^1^H NMR (500 MHz, CDCl_3_): 7.51–7.29 (m, 5H), 7.55 (t, *J* = 7.5 Hz, 1H), 6.28 (br s, 2H), 3.84 (s, 3H); ^13^C NMR (125 MHz, CDCl_3_): 164.8, 137.3, 129.4, 129.4, 129.2, 128.8, 52.4.

*Ethyl 2-hydrazono-3-phenylpropanoate* (**4i**). ^1^H NMR (500 MHz, CDCl_3_), *E*-isomer (major): 7.32–7.20 (m, 5H), 6.04 (br s, 2H), 4.33 (q, *J* = 7.0 Hz, 2H), 3,90 (s, 2H), 1.37 (t, *J* = 7.0 Hz, 3H). *Z*-isomer (minor): 8.16 (br s, 2H), 7.32–7.20 (m, 5H), 4.14 (q, *J* = 7.0 Hz, 2H), 3,69 (s, 2H), 1.22 (t, *J* = 7.0 Hz, 3H); ^13^C NMR (125 MHz, CDCl_3_), *E*-isomer (major): 165.2, 138.1, 134.8, 129.0, 128.0, 126.9, 61.5, 30.5, 14.3. *Z*-isomer (minor): 162.7, 139.2, 130.4, 128.8, 128.1, 126.1, 60.2, 39.5, 14.0.

Copies of the ^1^H and ^13^C NMR spectra of all products can be found in the Supplementary Materials.

## 4. Conclusions

In summary, we presented a novel and useful transfer hydrogenation methodology for the reduction of the carbene moiety of α-diazocarbonyl compounds into a methylene group catalyzed by recyclable and reusable supported Au nanoparticles on TiO_2_. The reductant can be readily available NH_3_BH_3_ or NaBH_4_. The characteristic of the reduction protocol is that one stoichiometric hydride equivalent from the boron hydride reagent and one proton that arises from the protic solvent (methanol) are required. In case of using an excess of reducing agent, a side catalytic pathway operates that reduces the α-diazocarbonyl compounds into the corresponding hydrazones. This work exemplifies the unique catalytic behavior of Au nanoparticles relative to other supported metallic noble metals such as Pt [20], where the reductive pathway of the formation of hydrazones occurs exclusively.

## Data Availability

Data is contained within the article or Supplementary Materials.

## Supplementary Materials

Copies of ^1^H and ^13^C NMR of reactants and products are available online at https://www.mdpi.com/2079-4991/11/1/248/s1.
