# Supplementary material for: Reduction of the Diazo Functionality of α-Diazocarbonyl Compounds into a Methylene Group by NH3BH3 or NaBH4 Catalyzed by Au Nanoparticles
Source: Nanomaterials (Basel). 2021 Jan 18;11(1):248. doi: 10.3390/nano11010248 (PMC7832297; doi:10.3390/nano11010248)

SUPPORTING INFORMATION

Reduction of the diazo functionality of α-diazocarbonyl compounds into a methylene group by NH_3BH_3 or $NaBH_4$ catalyzed by Au nanoparticles

Marios Kidonakis and Manolis Stratakis*

Department of Chemistry, University of Crete, Voutes 71003 Heraklion, Greece

stratakis@uoc.gr

^1H and ^{13}C NMR spectra of reactants

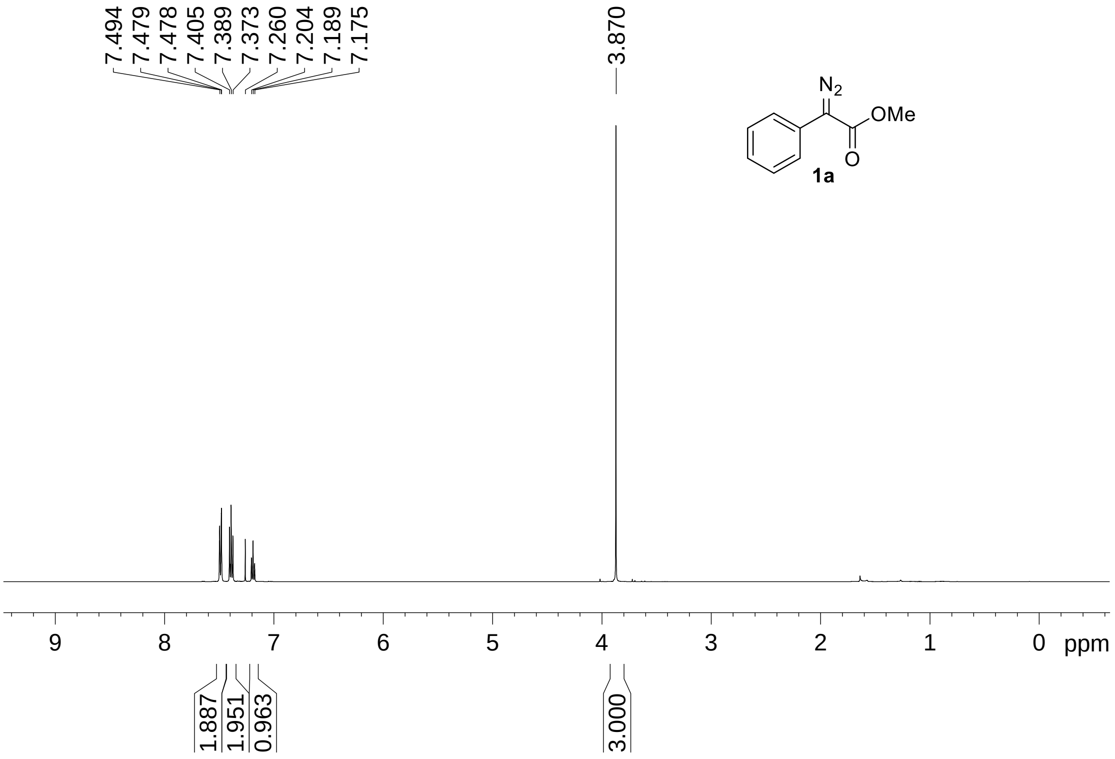

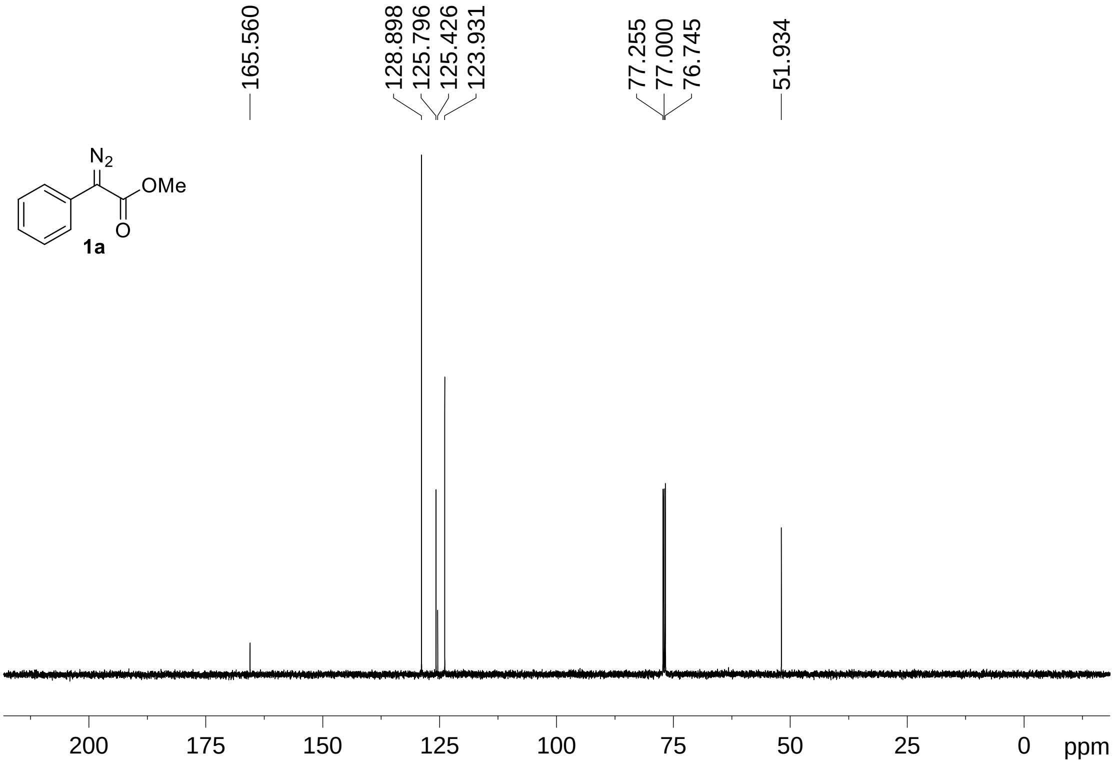

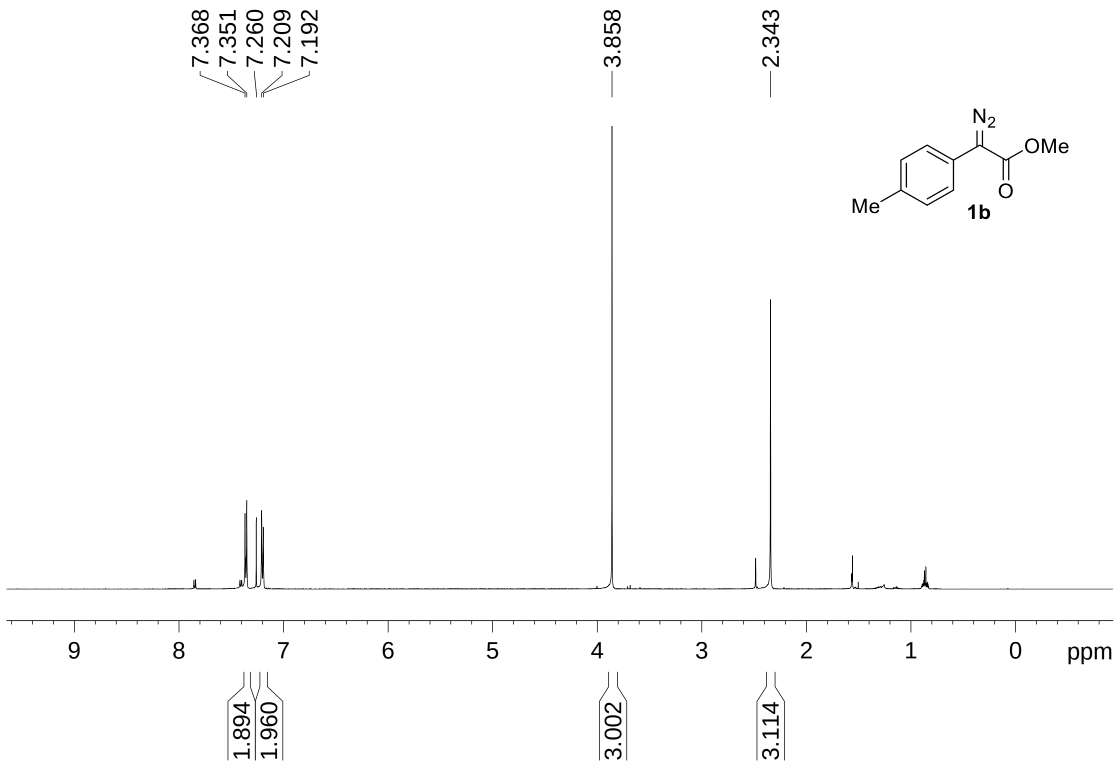
7.368
7.351
7.260
7.209
7.192
3.858
2.343
N2
OMe
O
Me
1b
9 8 7 6 5 4 3 2 1 0 ppm
1.894
1.960
3.002
3.114

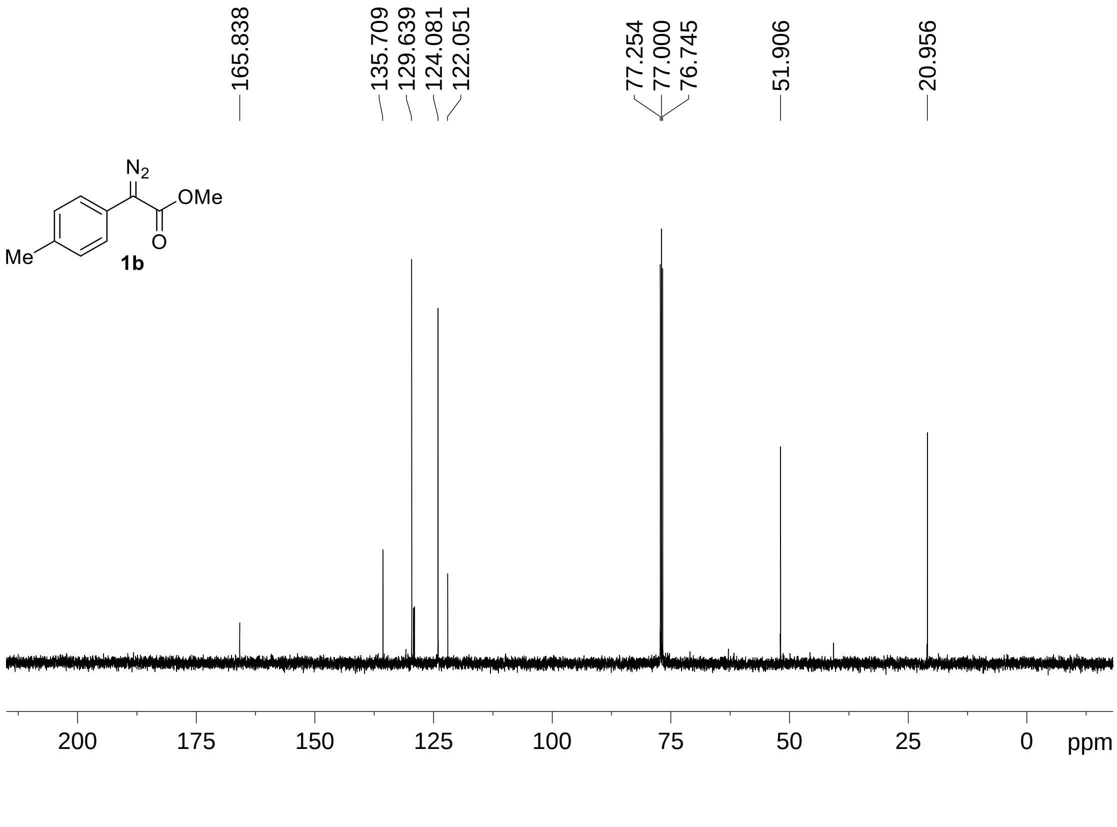
165.838
135.709
129.639
124.081
122.051
77.254
77.000
76.745
51.906
20.956
N2
OMe
O
Me
1b
200 175 150 125 100 75 50 25 0 ppm

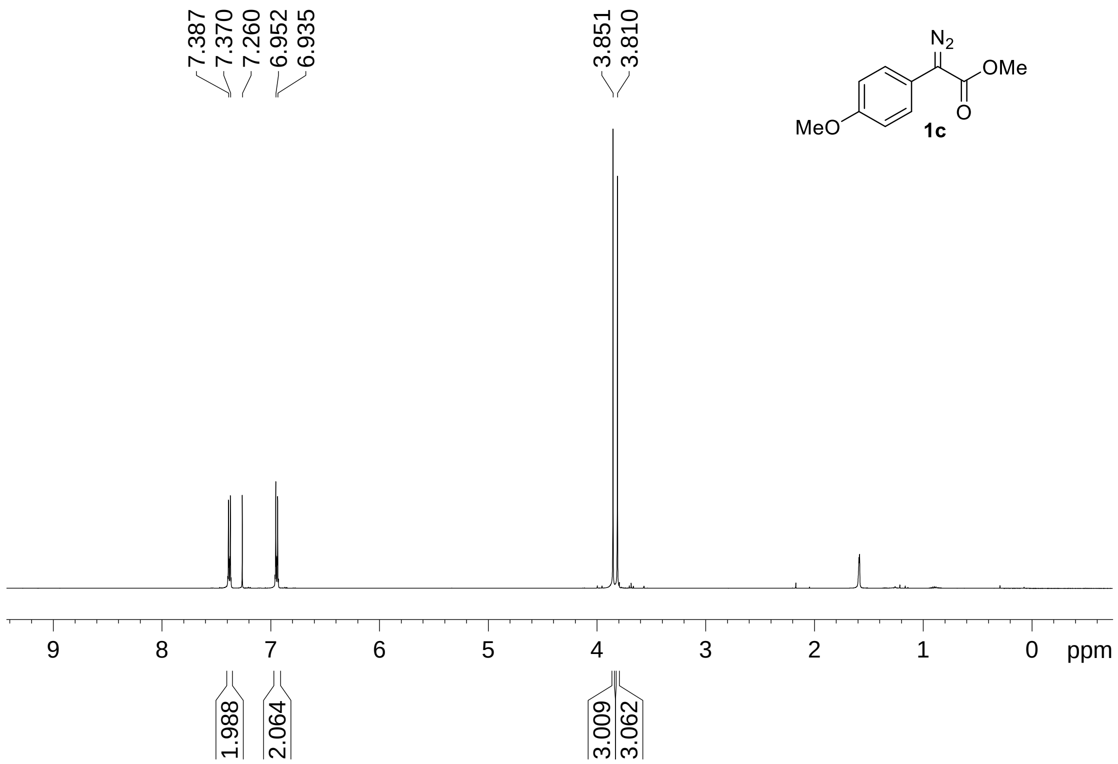
7.387
7.370
7.260
6.952
6.935
3.851
3.810
N2
OMe
O
MeO
1c
9 8 7 6 5 4 3 2 1 0 ppm
1.988
2.064
3.009
3.062

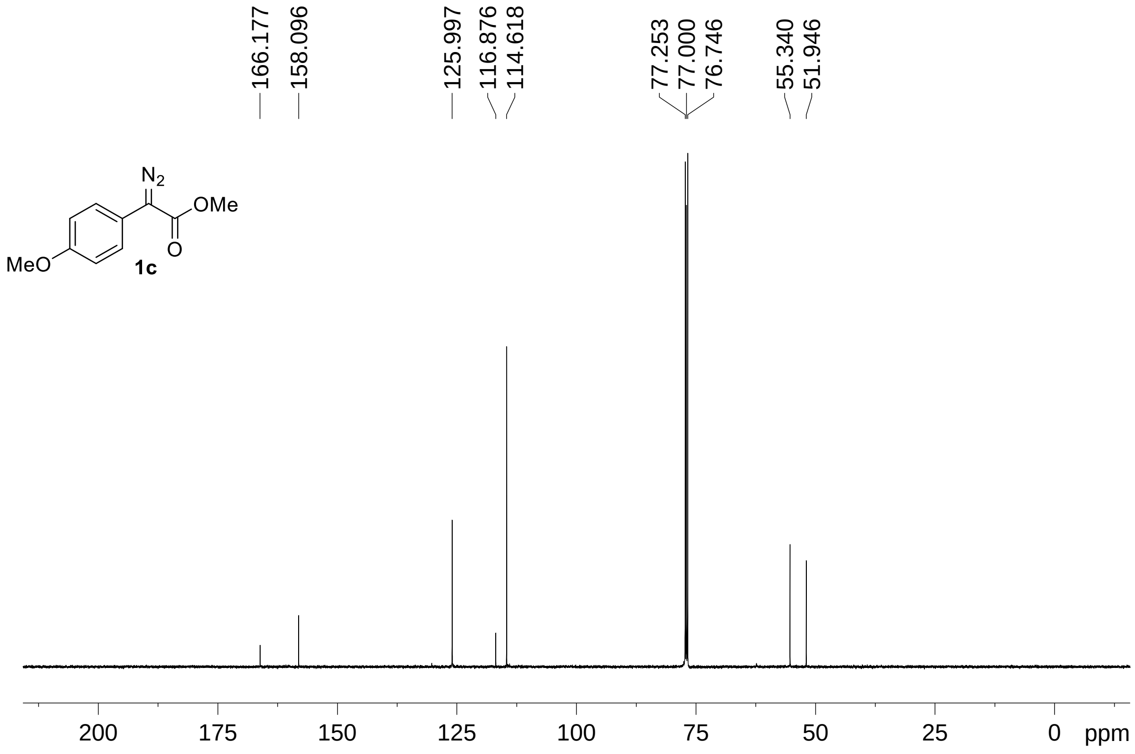
166.177
158.096
125.997
116.876
114.618
77.253
77.000
76.746
55.340
51.946
N2
OMe
O
MeO
1c
200 175 150 125 100 75 50 25 0 ppm

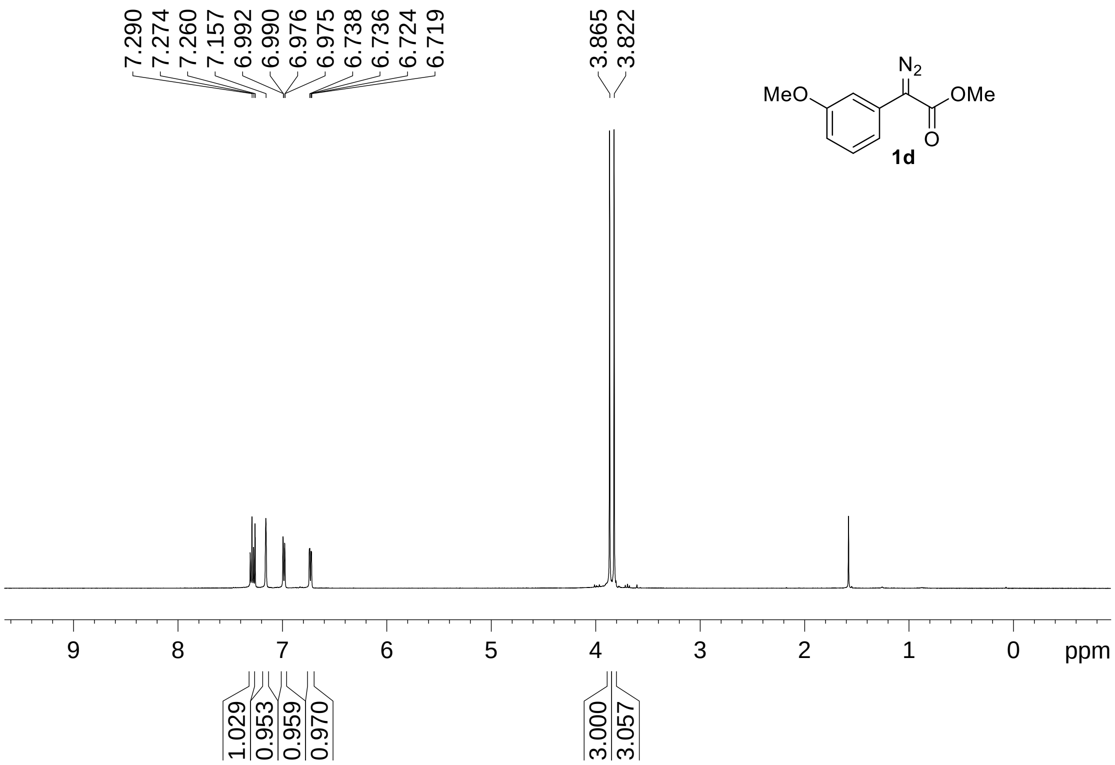
7.290
7.274
7.260
7.157
6.992
6.990
6.976
6.975
6.738
6.736
6.724
6.719
3.865
3.822
MeO
N2
OMe
O
1d
9
8
7
6
5
4
3
2
1
0
ppm
1.029
0.953
0.959
0.970
3.000
3.057

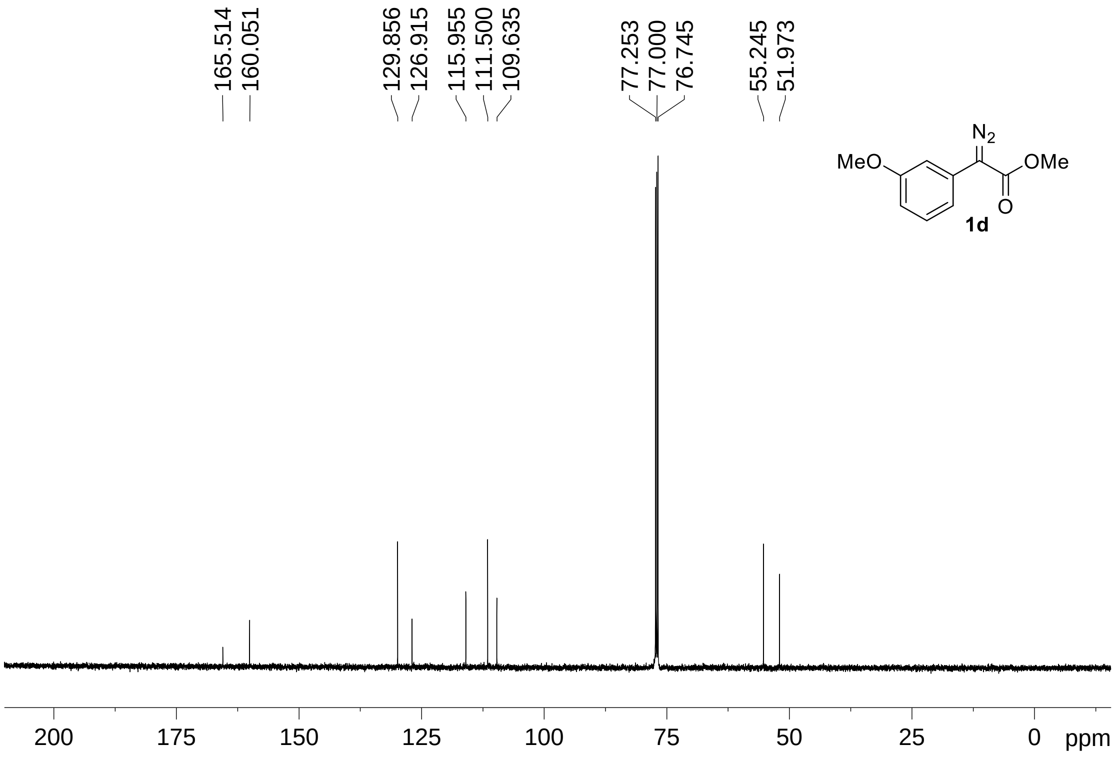
165.514
160.051
129.856
126.915
115.955
111.500
109.635
77.253
77.000
76.745
55.245
51.973
MeO
N2
OMe
O
1d
200
175
150
125
100
75
50
25
0
ppm

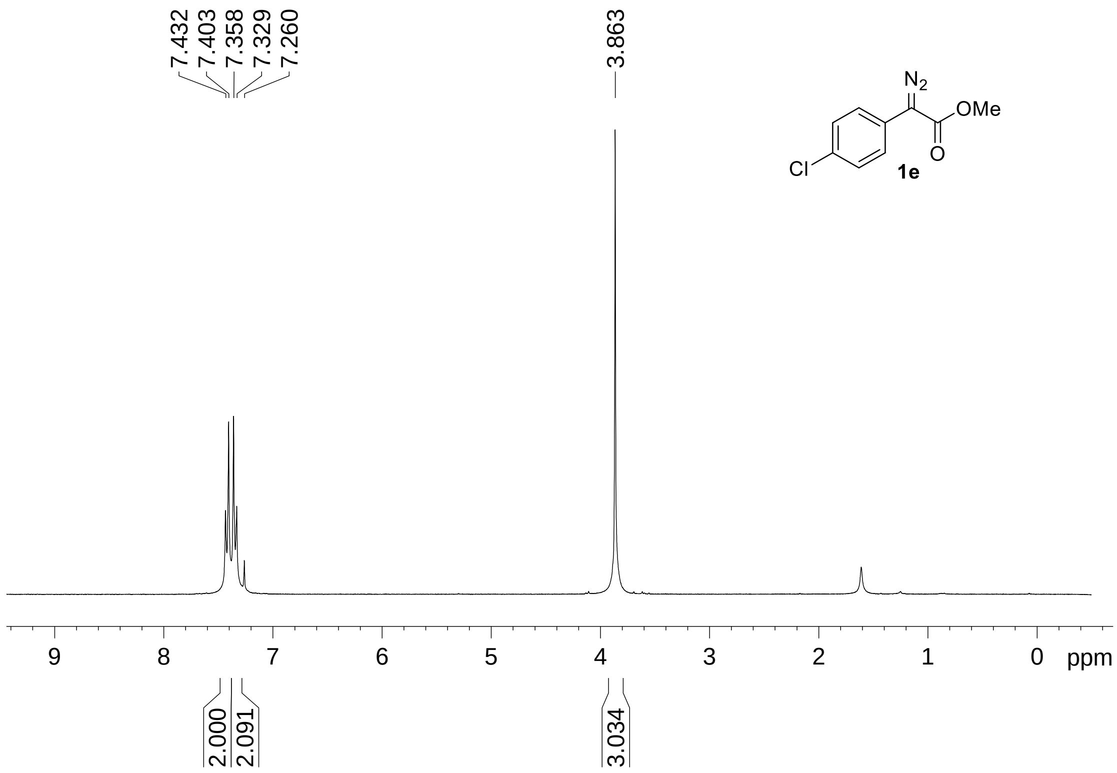
7.432
7.403
7.358
7.329
7.260
3.863
N2
OMe
O
Cl
1e
9
8
7
6
5
4
3
2
1
0
ppm
2.000
2.091
3.034

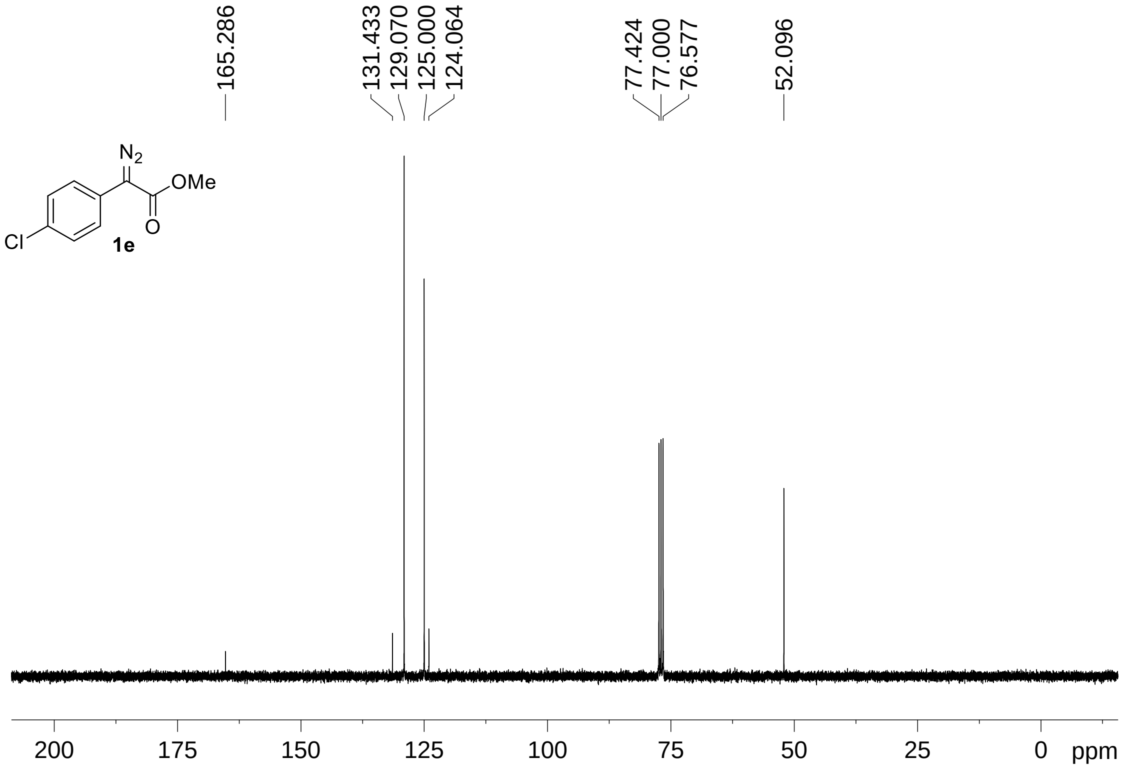
165.286
131.433
129.070
125.000
124.064
77.424
77.000
76.577
52.096
N2
OMe
O
Cl
1e
200
175
150
125
100
75
50
25
0
ppm

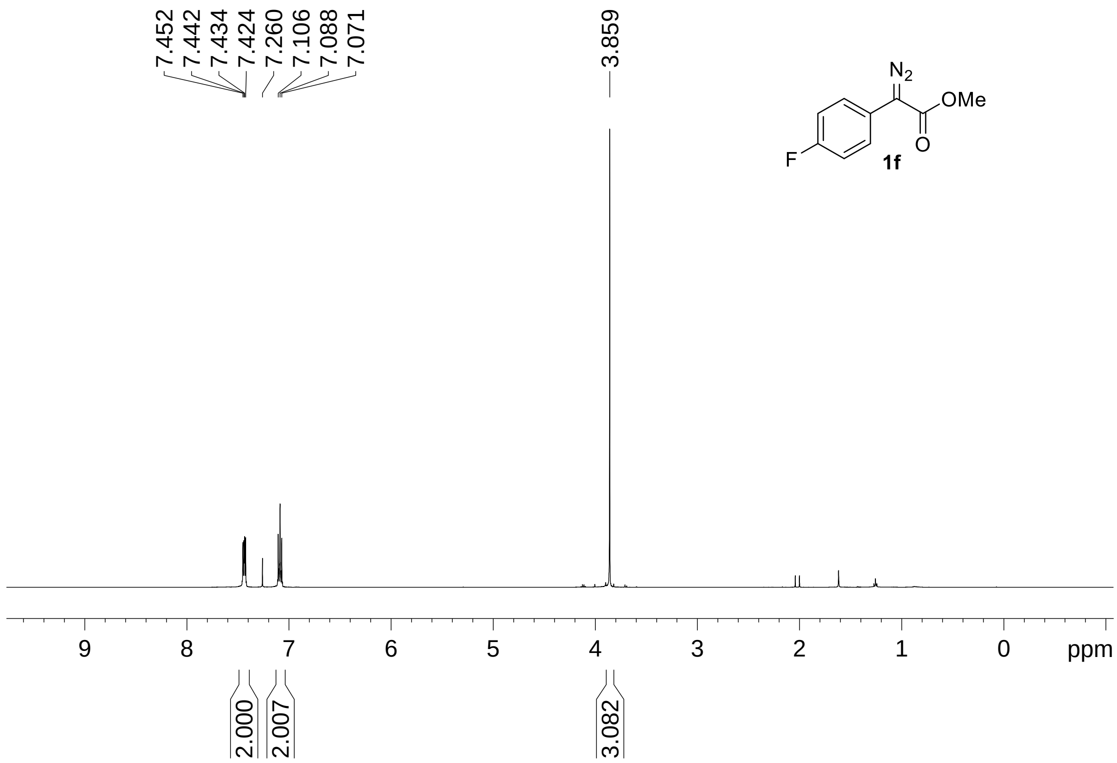
7.452
7.442
7.434
7.424
7.260
7.106
7.088
7.071
3.859
N2
OMe
F
O
1f
9 8 7 6 5 4 3 2 1 0 ppm
2.000
2.007
3.082

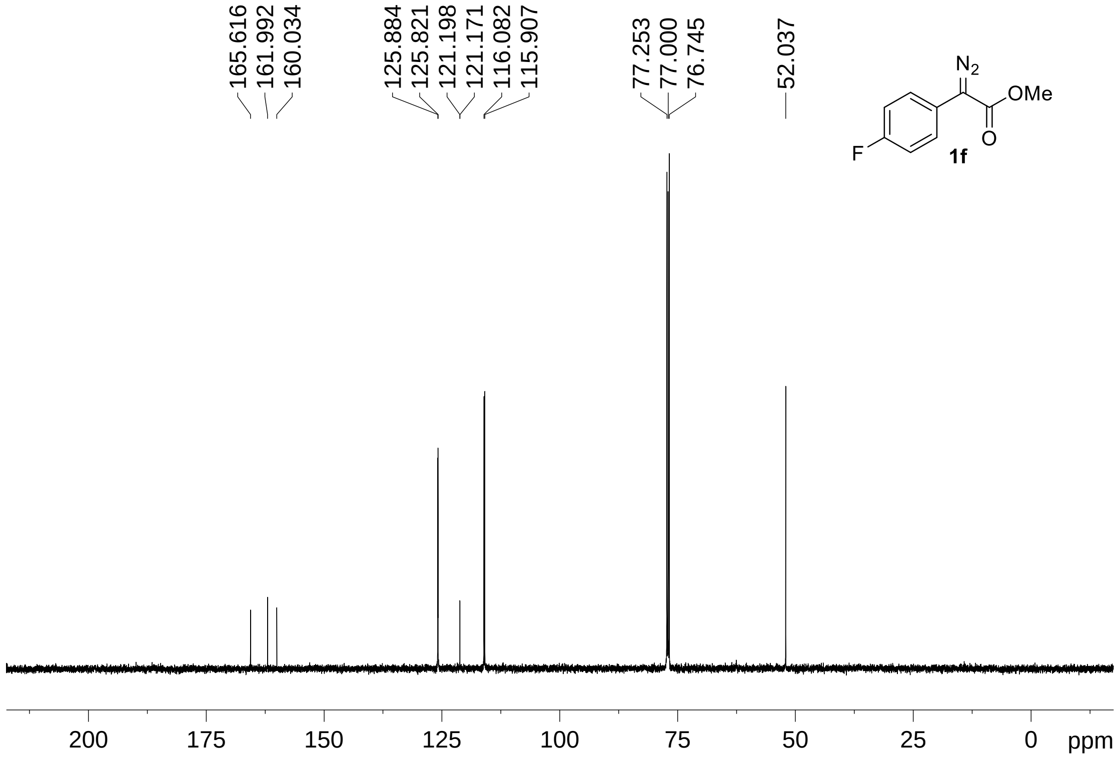
165.616
161.992
160.034
125.884
125.821
121.198
121.171
116.082
115.907
77.253
77.000
76.745
52.037
N2
OMe
F
O
1f
200 175 150 125 100 75 50 25 0 ppm

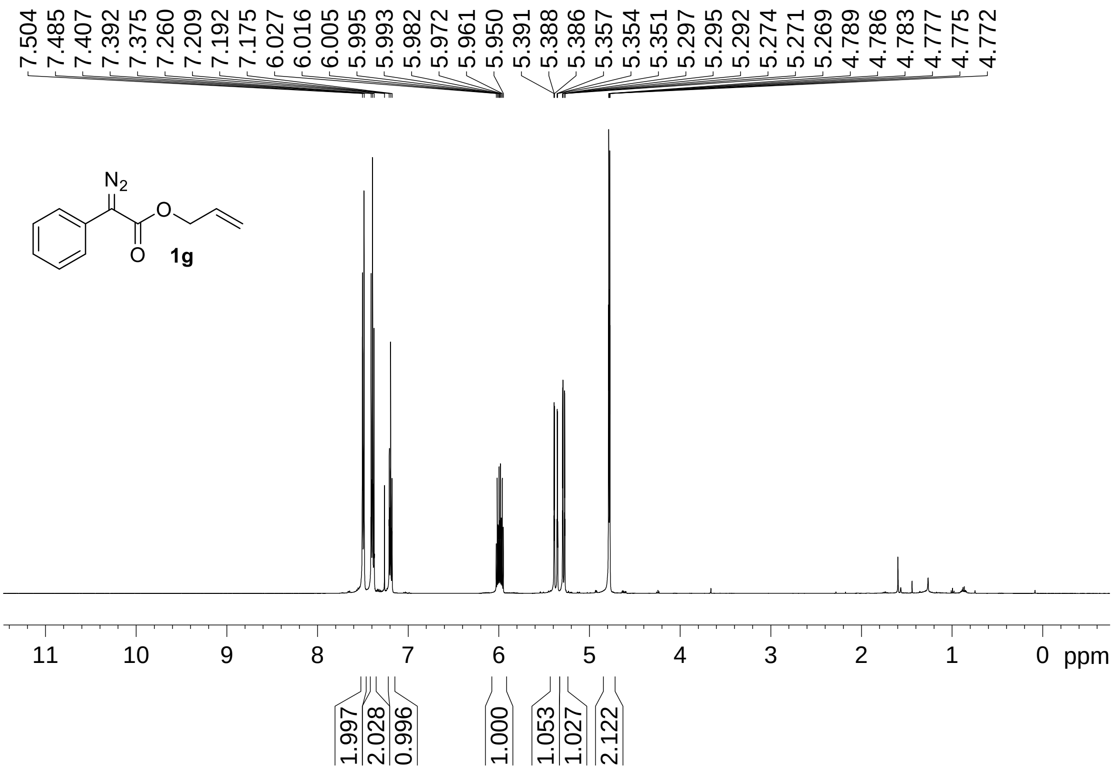

7.504
7.485
7.407
7.392
7.375
7.260
7.209
7.192
7.175
6.027
6.016
6.005
5.995
5.993
5.982
5.972
5.961
5.950
5.391
5.388
5.386
5.357
5.354
5.351
5.297
5.295
5.292
5.274
5.271
5.269
4.789
4.786
4.783
4.777
4.775
4.772
N2
O
1g
11 10 9 8 7 6 5 4 3 2 1 0 ppm
1.997
2.028
0.996
1.000
1.053
1.027
2.122

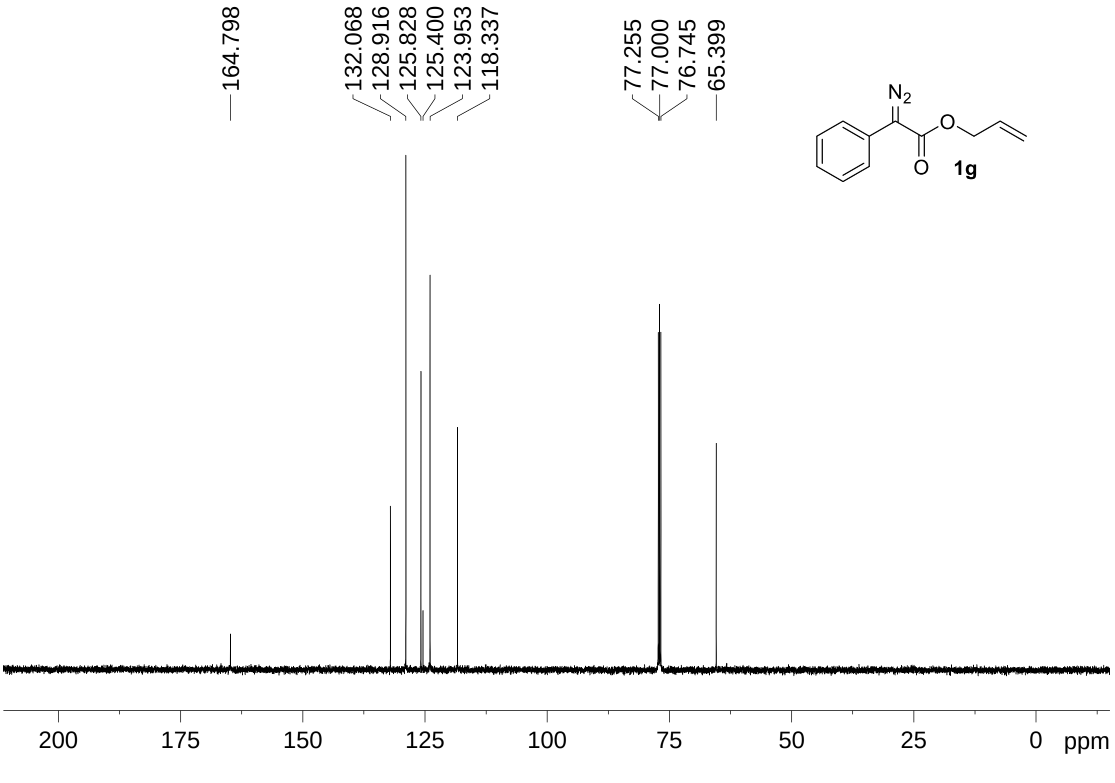

164.798
132.068
128.916
125.828
125.400
123.953
118.337
77.255
77.000
76.745
65.399
N2
O
1g
200 175 150 125 100 75 50 25 0 ppm

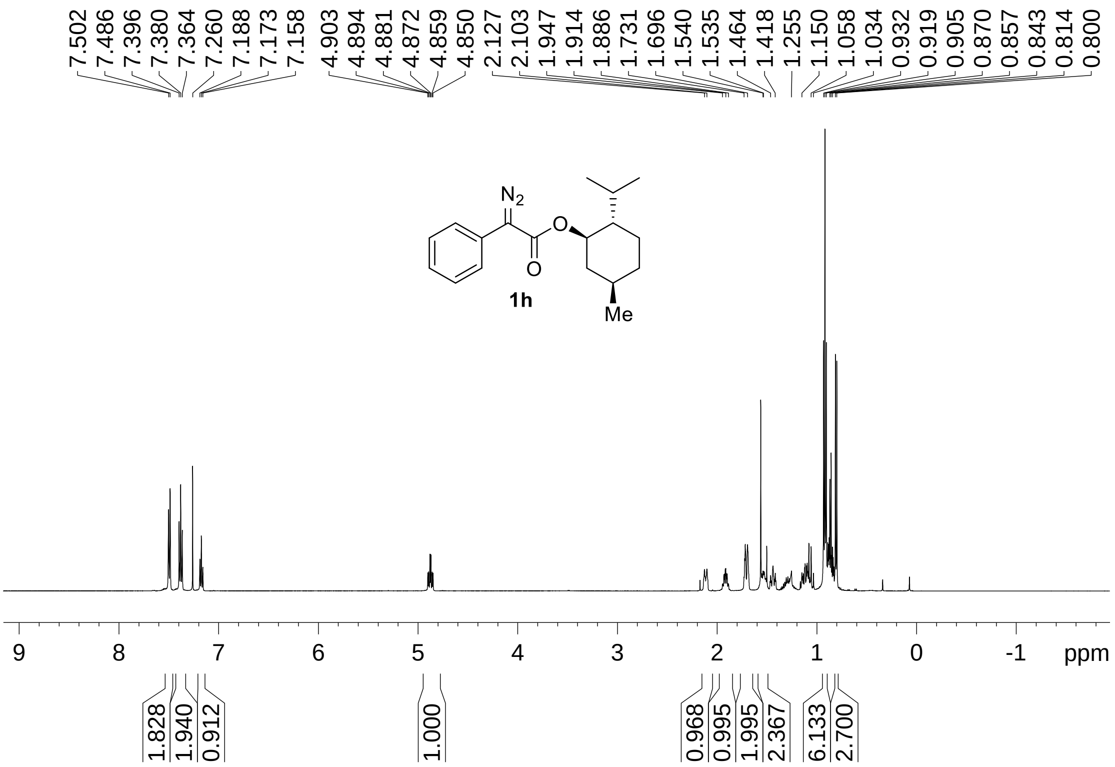
7.502
7.486
7.396
7.380
7.364
7.260
7.188
7.173
7.158
4.903
4.894
4.881
4.872
4.859
4.850
2.127
2.103
1.947
1.914
1.886
1.731
1.696
1.540
1.535
1.464
1.418
1.255
1.150
1.058
1.034
0.932
0.919
0.905
0.870
0.857
0.843
0.814
0.800
N2
O
O
1h
Me
9
8
7
6
5
4
3
2
1
0
-1
ppm
1.828
1.940
0.912
1.000
0.968
0.995
1.995
2.367
6.133
2.700

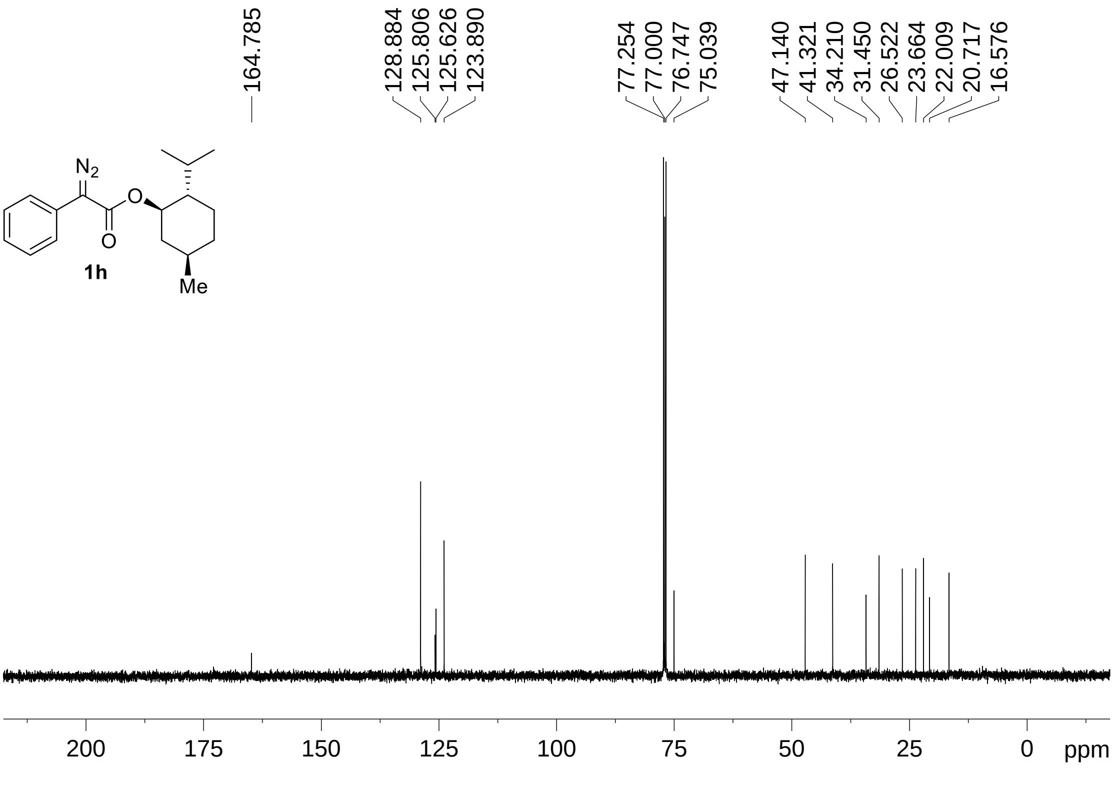
164.785
128.884
125.806
125.626
123.890
77.254
77.000
76.747
75.039
47.140
41.321
34.210
31.450
26.522
23.664
22.009
20.717
16.576
N2
O
O
1h
Me
200
175
150
125
100
75
50
25
0
ppm

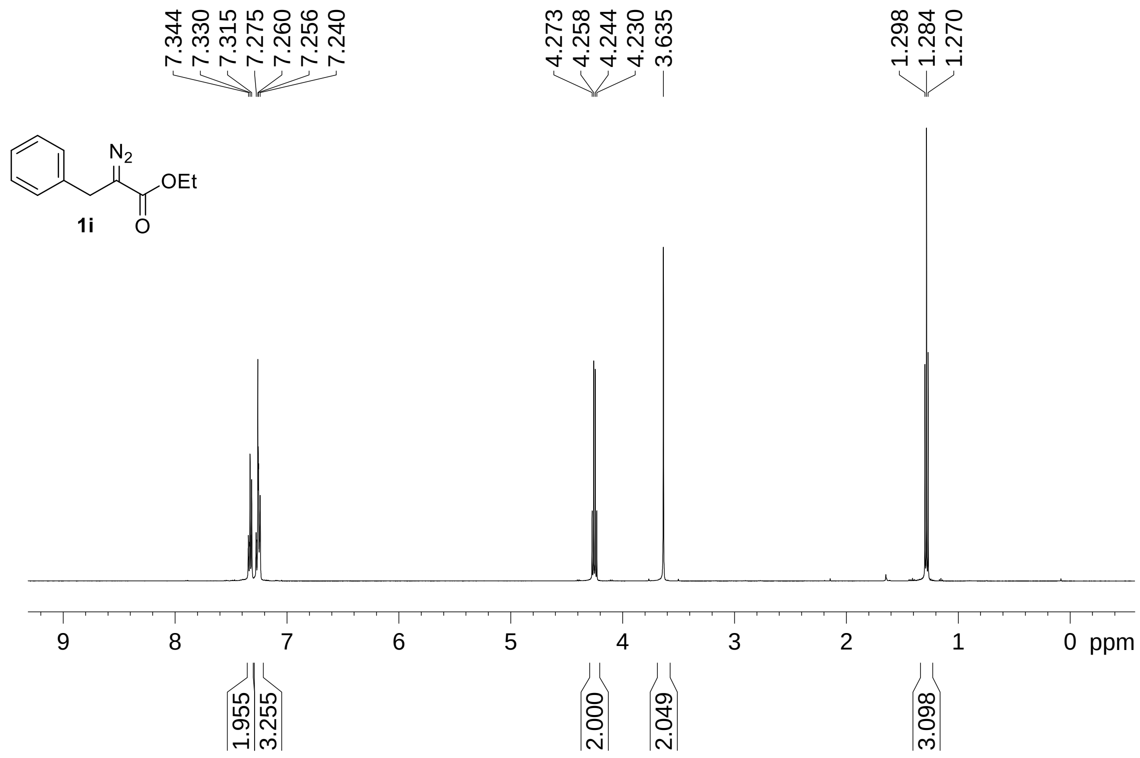
7.344
7.330
7.315
7.275
7.260
7.256
7.240
4.273
4.258
4.244
4.230
3.635
1.298
1.284
1.270
N2
OEt
O
1i
9 8 7 6 5 4 3 2 1 0 ppm
1.955
3.255
2.000
2.049
3.098

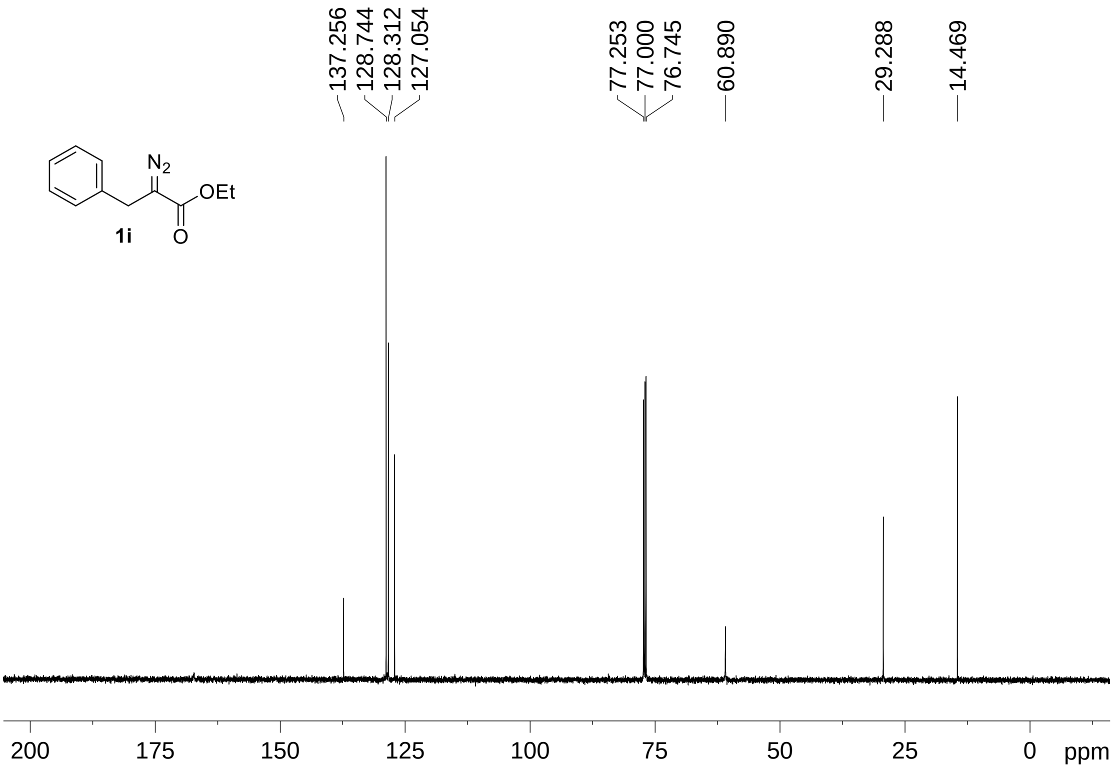
137.256
128.744
128.312
127.054
77.253
77.000
76.745
60.890
29.288
14.469
N2
OEt
O
1i
200 175 150 125 100 75 50 25 0 ppm

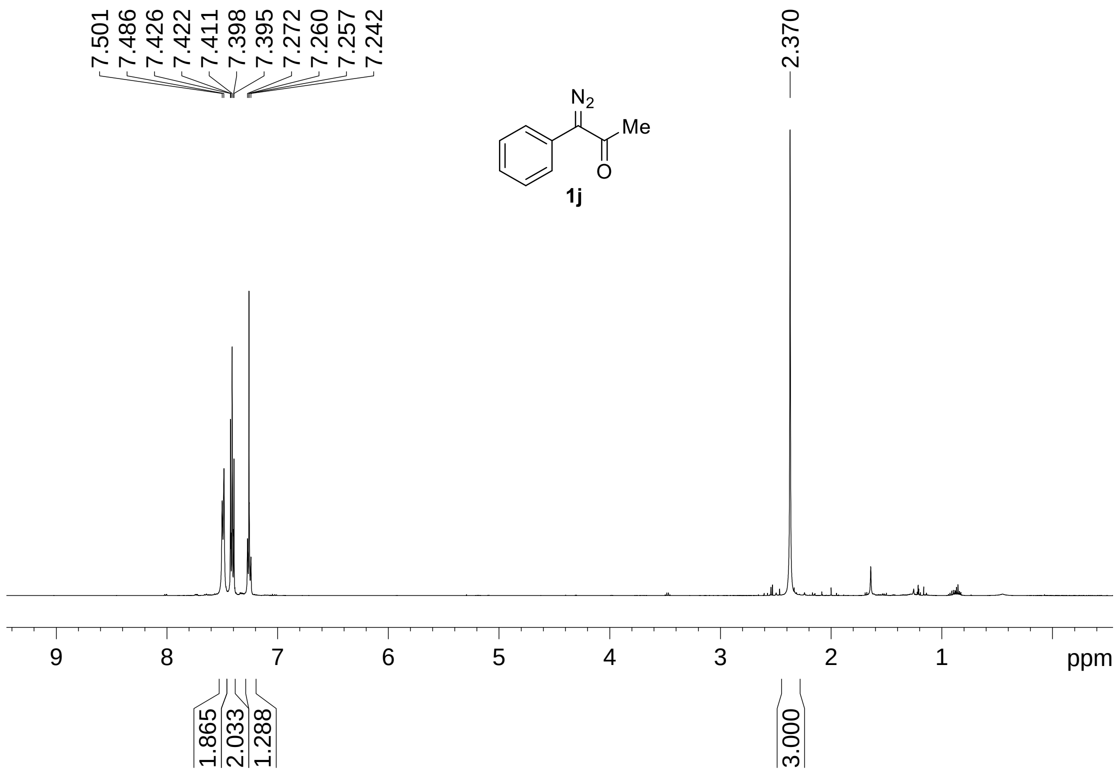
7.501
7.486
7.426
7.422
7.411
7.398
7.395
7.272
7.260
7.257
7.242
2.370
N_2
Me
O
1j
9
8
7
6
5
4
3
2
1
ppm
1.865
2.033
1.288
3.000

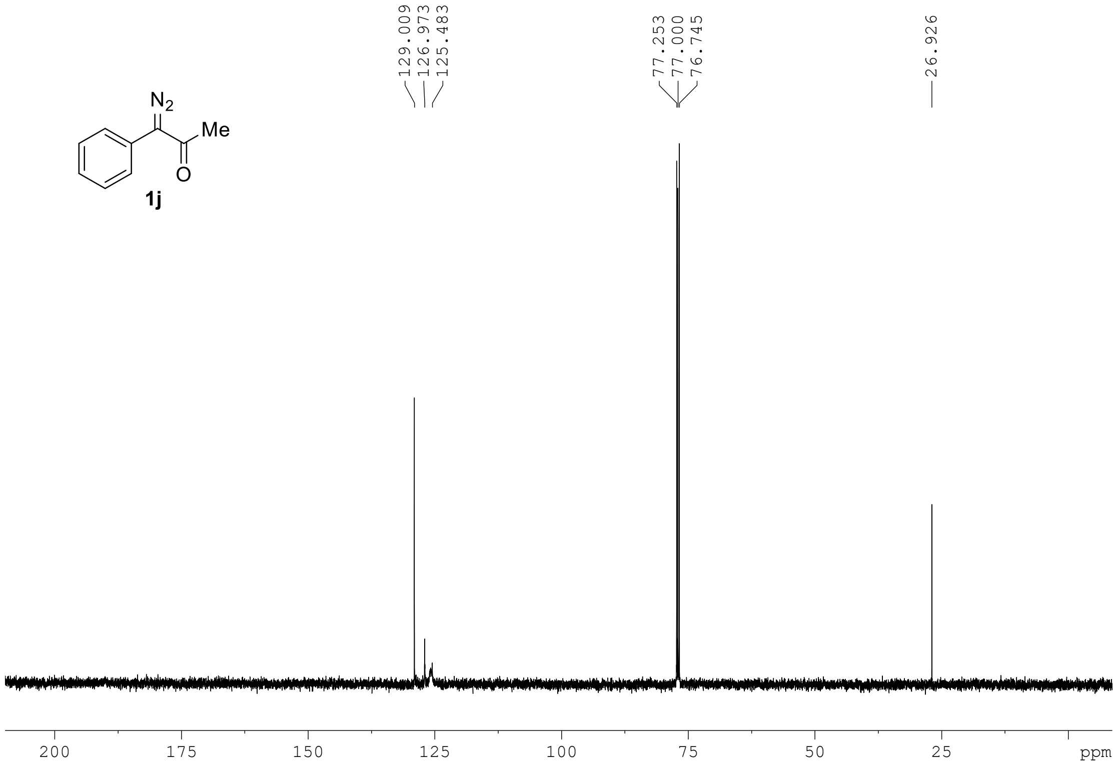
129.009
126.973
125.483
77.253
77.000
76.745
26.926
N_2
Me
O
1j
200
175
150
125
100
75
50
25
ppm

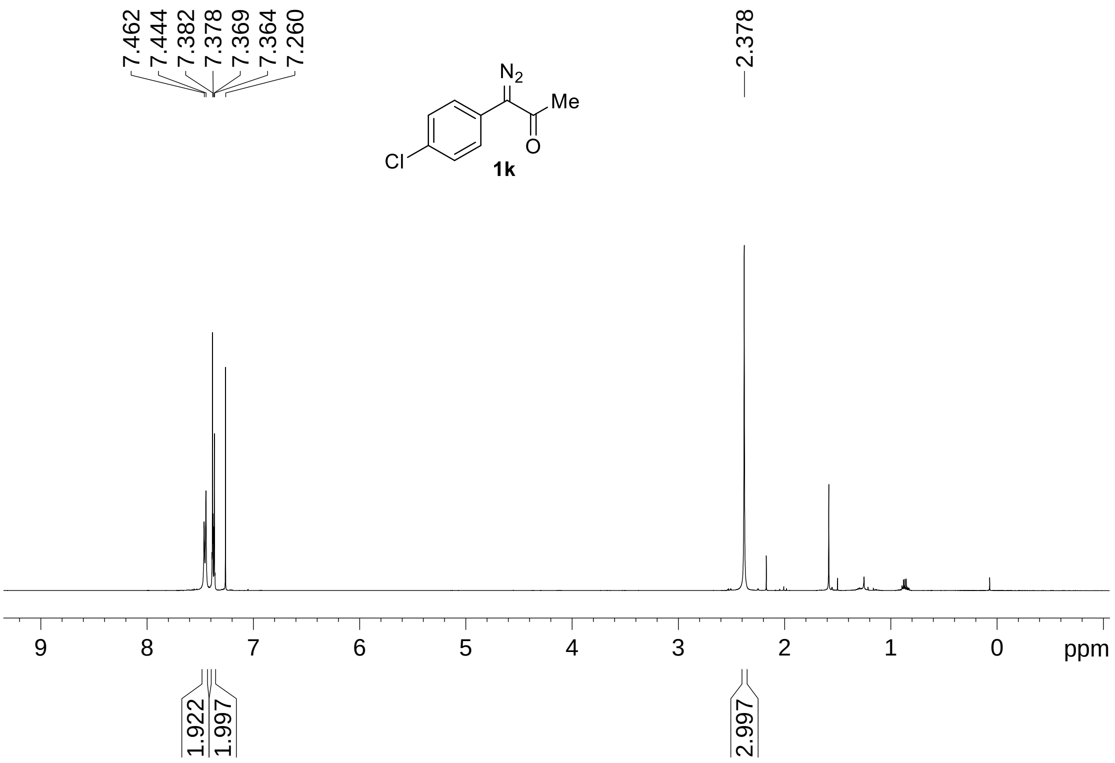
7.462
7.444
7.382
7.378
7.369
7.364
7.260
2.378
N2
Me
O
Cl
1k
9
8
7
6
5
4
3
2
1
0
ppm
1.922
1.997
2.997

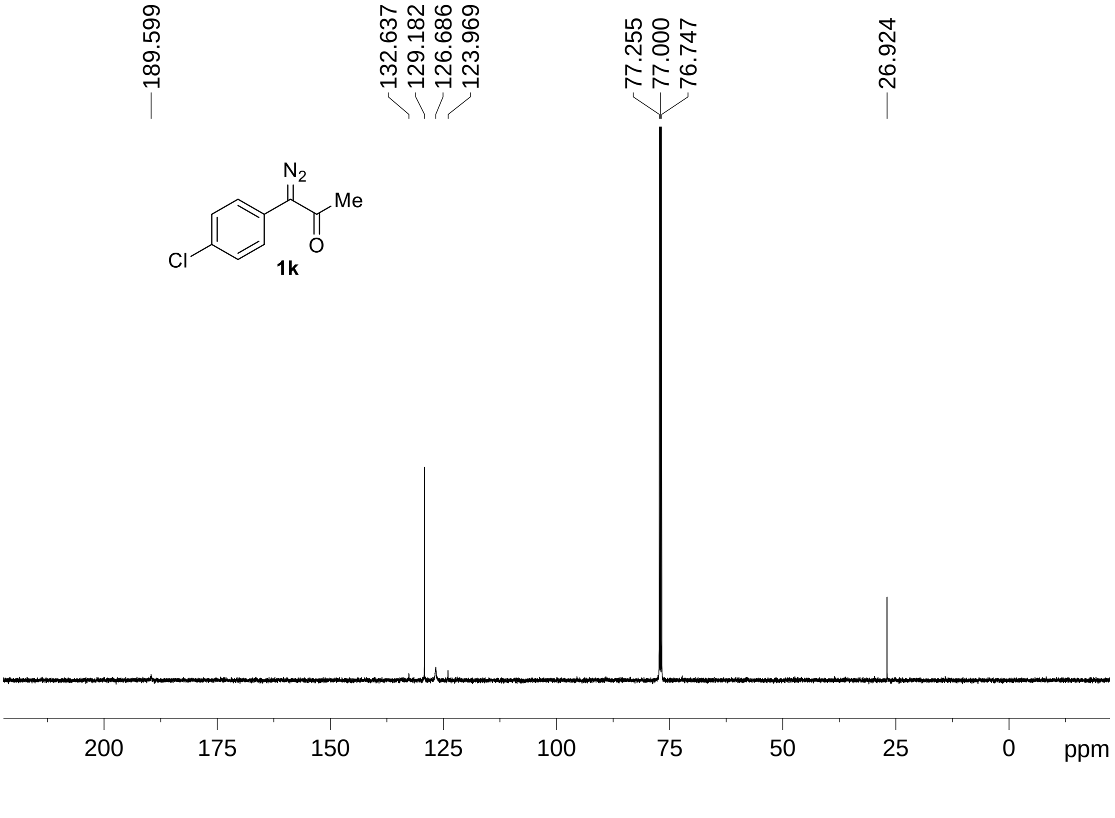
189.599
132.637
129.182
126.686
123.969
77.255
77.000
76.747
26.924
N2
Me
O
Cl
1k
200
175
150
125
100
75
50
25
0
ppm

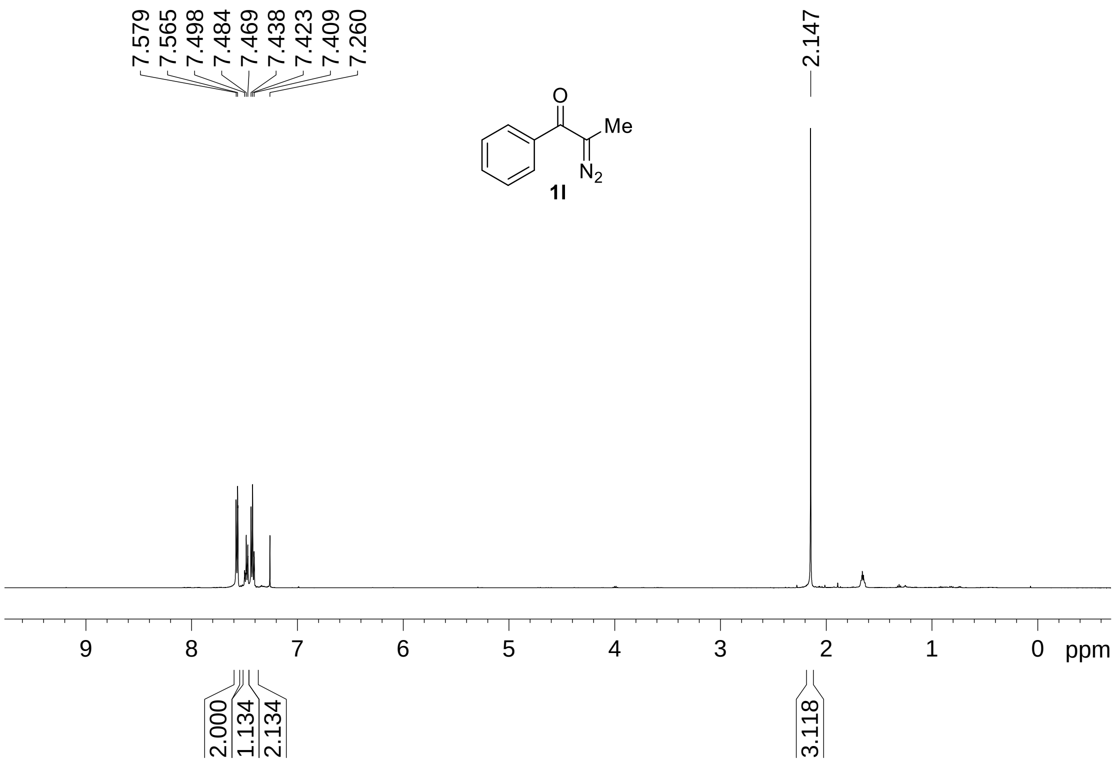
7.579
7.565
7.498
7.484
7.469
7.438
7.423
7.409
7.260
2.147
O
Me
N2
1l
9
8
7
6
5
4
3
2
1
0
ppm
2.000
1.134
2.134
3.118

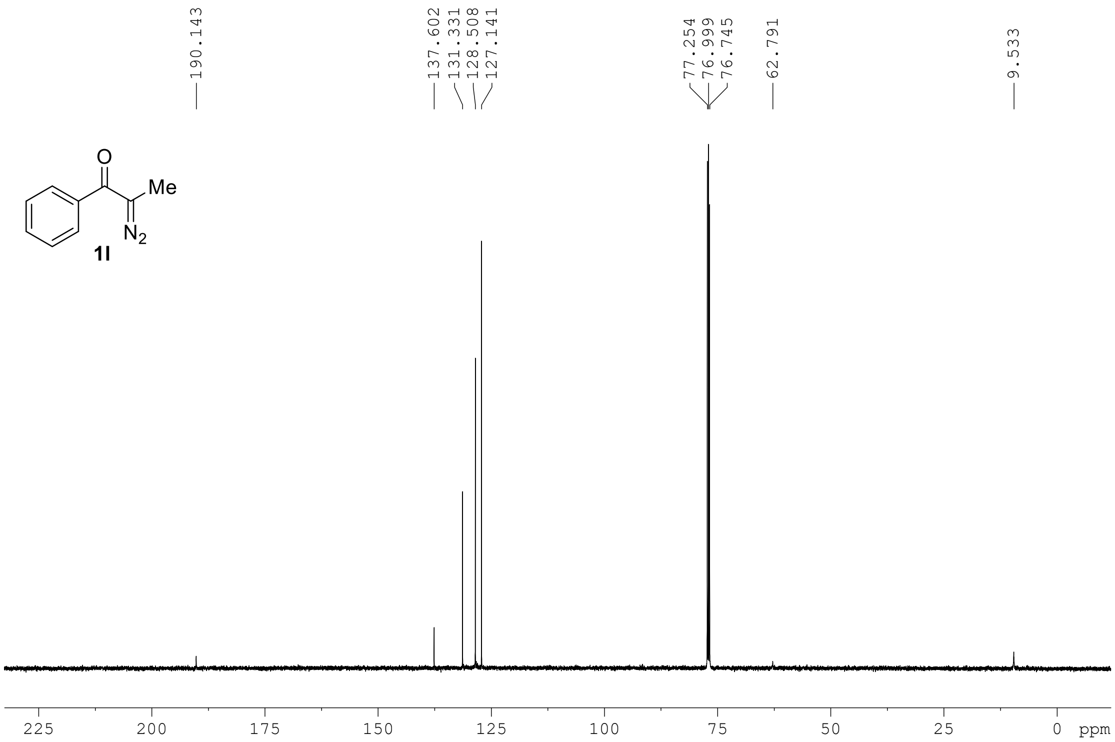
190.143
137.602
131.331
128.508
127.141
77.254
76.999
76.745
62.791
9.533
O
Me
N2
1l
225
200
175
150
125
100
75
50
25
0
ppm

^{1}H and ^{13}C NMR spectra of products

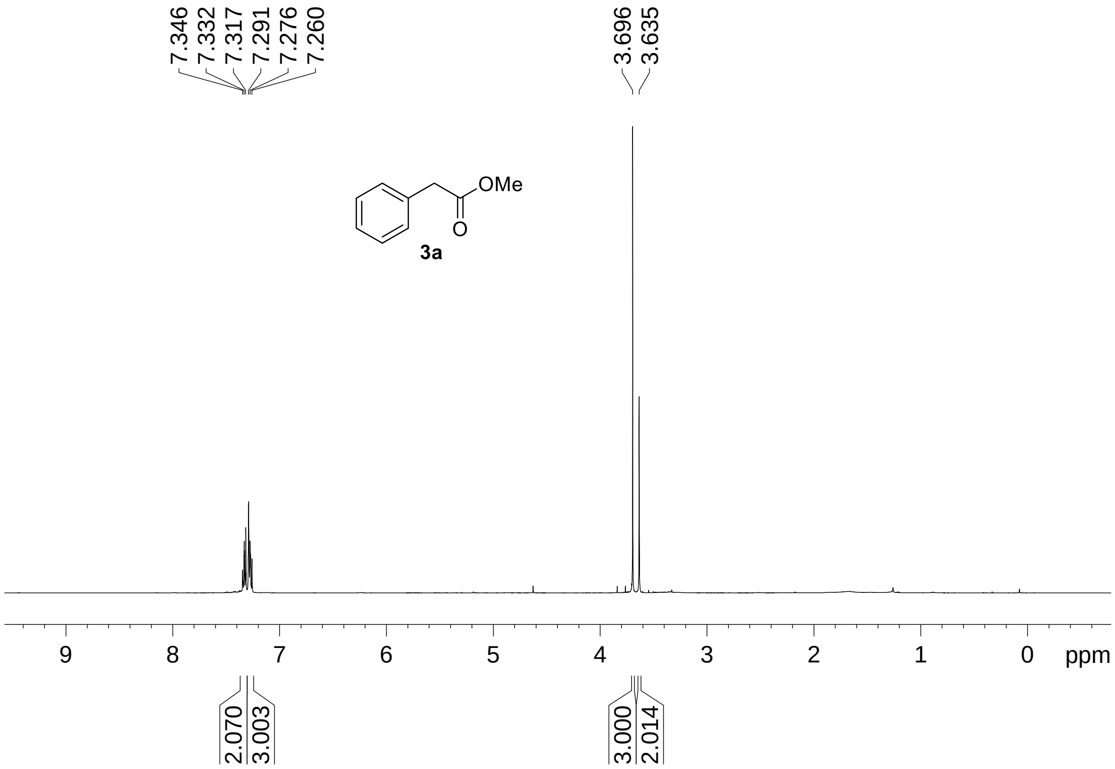

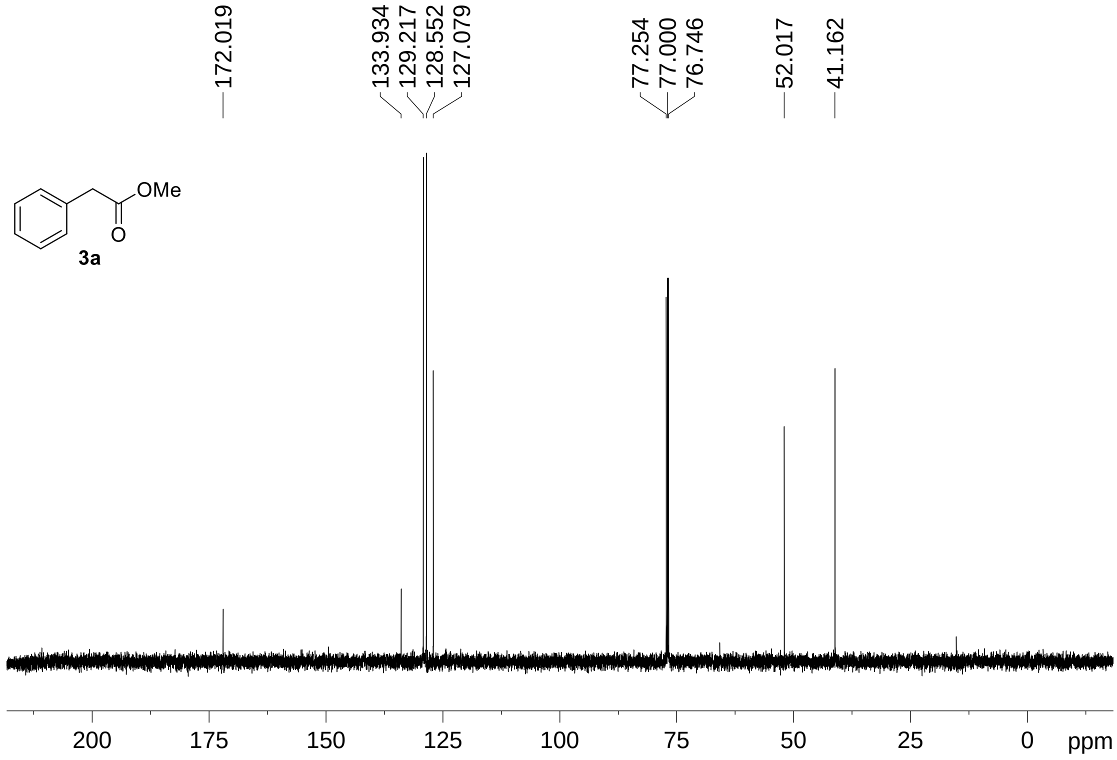

Reaction of α-diazocarbonyl compound 1a with 1 molar equiv of $NaBH_4$

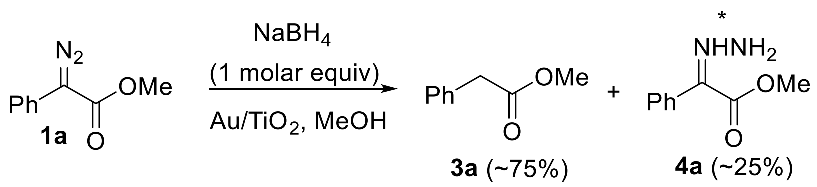

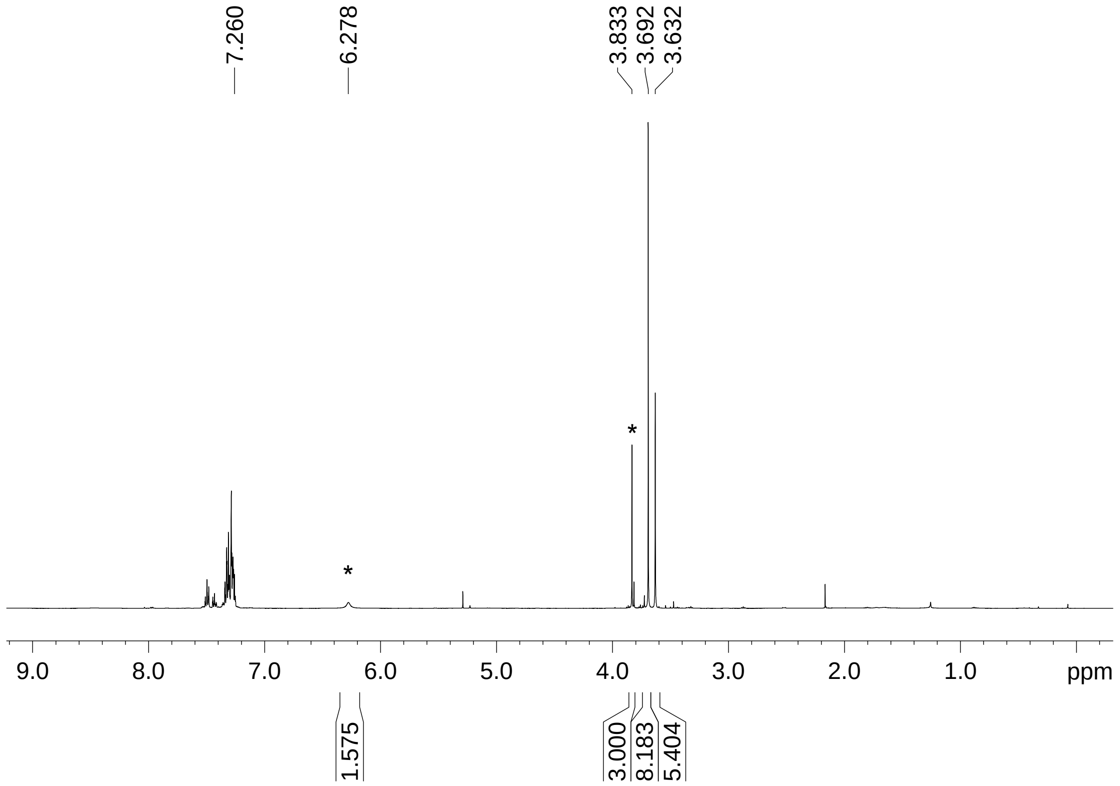

*Signals correspond to hydrazone **4a**

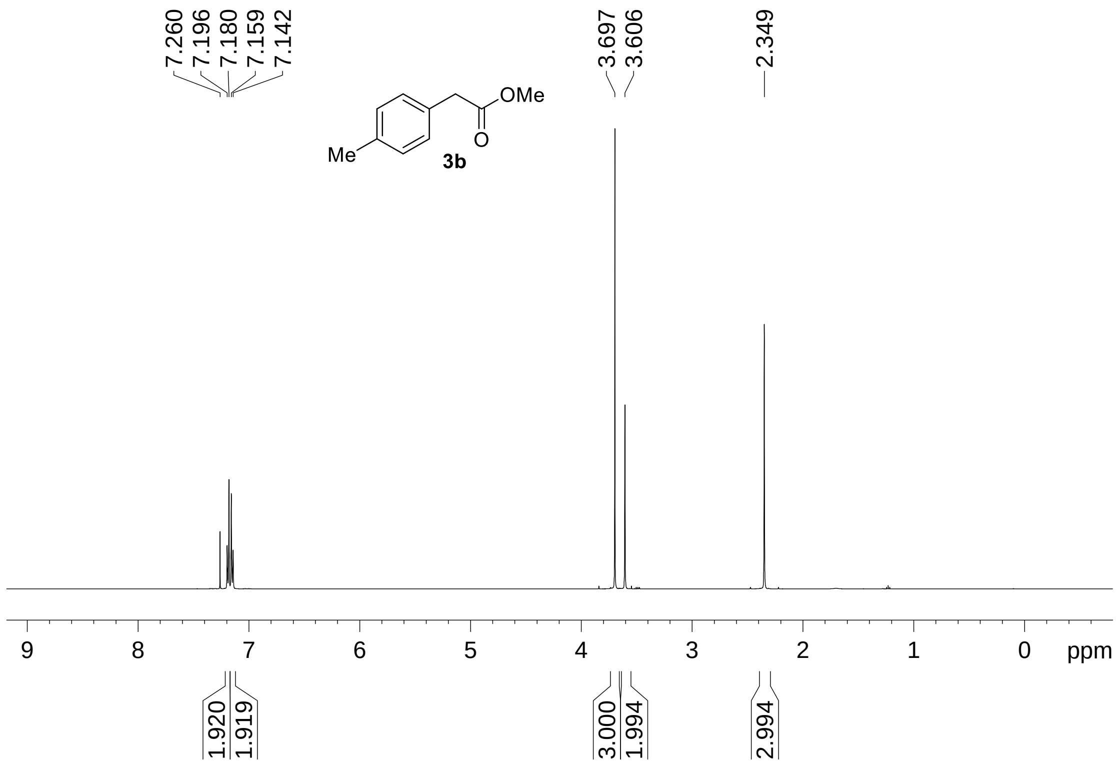
7.260
7.196
7.180
7.159
7.142
3.697
3.606
2.349
OMe
O
Me
3b
9 8 7 6 5 4 3 2 1 0 ppm
1.920
1.919
3.000
1.994
2.994

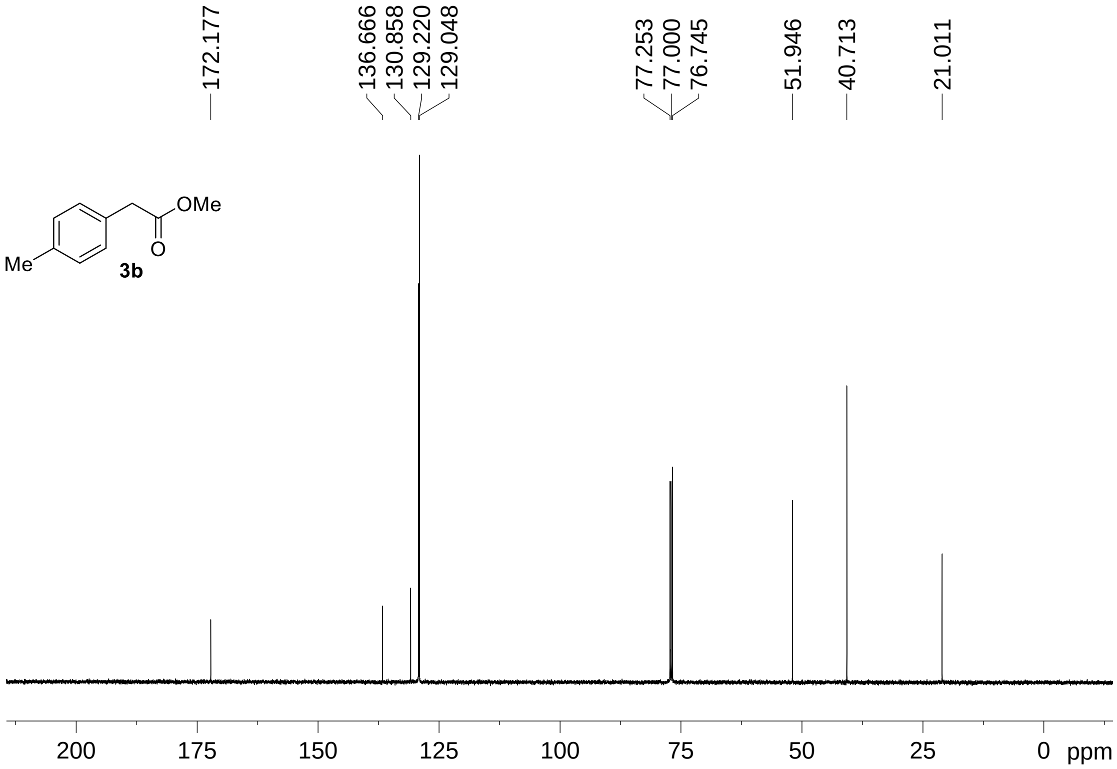
172.177
136.666
130.858
129.220
129.048
77.253
77.000
76.745
51.946
40.713
21.011
OMe
O
Me
3b
200 175 150 125 100 75 50 25 0 ppm

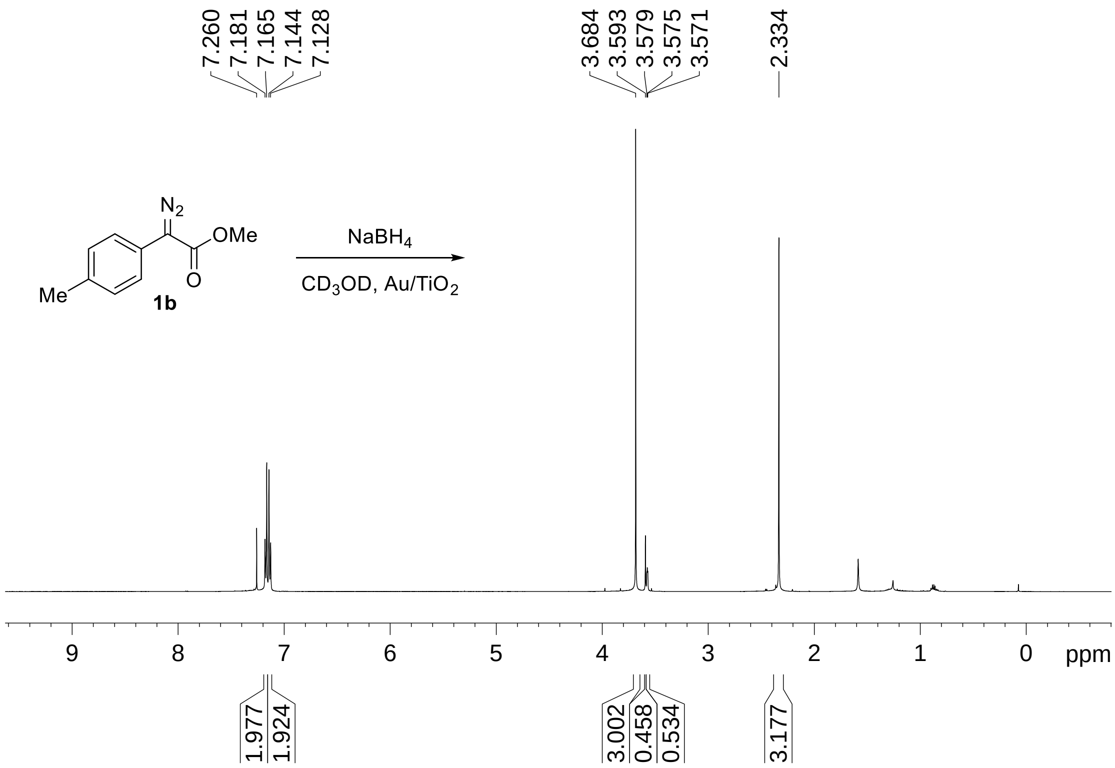
7.260
7.181
7.165
7.144
7.128
3.684
3.593
3.579
3.575
3.571
2.334
N_2
OMe
O
Me
1b
$NaBH_4$
CD_3OD, Au/TiO_2
9
8
7
6
5
4
3
2
1
0
ppm
1.977
1.924
3.002
0.458
0.534
3.177

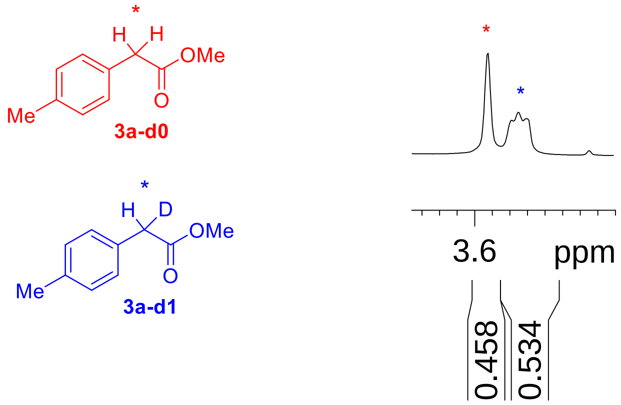
H
H
OMe
O
Me
3a-d0
H
D
OMe
O
Me
3a-d1
3.6
ppm
0.458
0.534

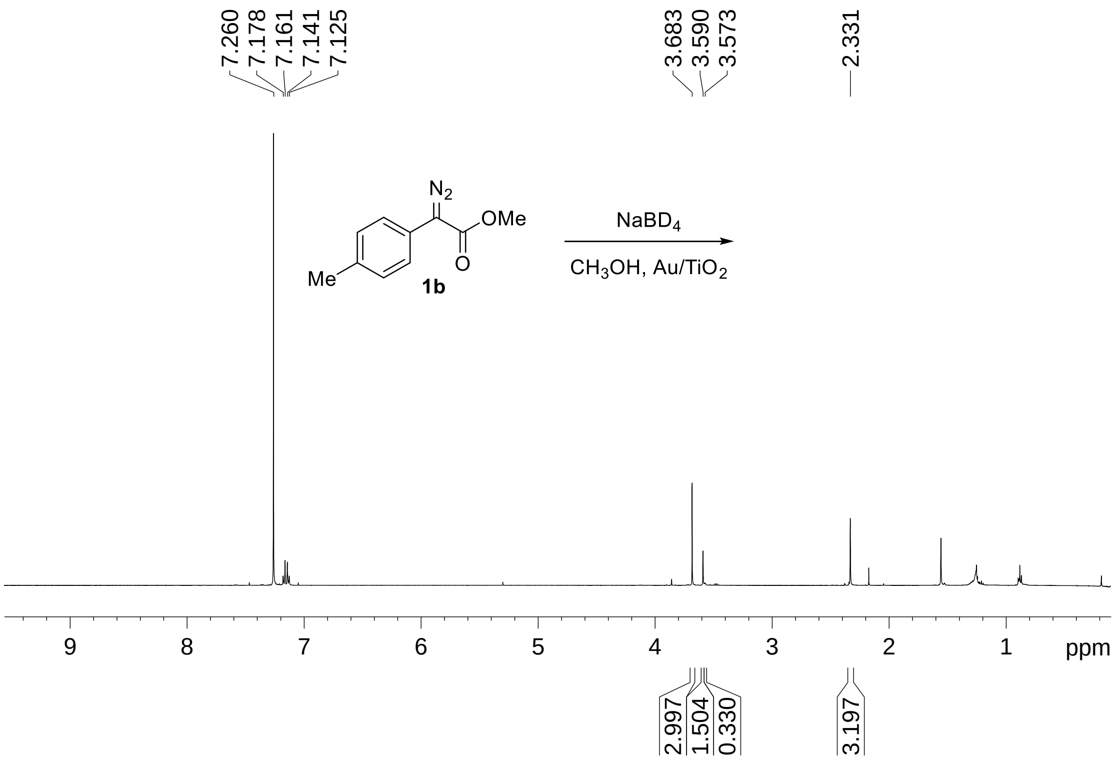
7.260
7.178
7.161
7.141
7.125
3.683
3.590
3.573
2.331
N2
OMe
Me
O
1b
NaBD4
CH3OH, Au/TiO2
9
8
7
6
5
4
3
2
1
ppm
2.997
1.504
0.330
3.197

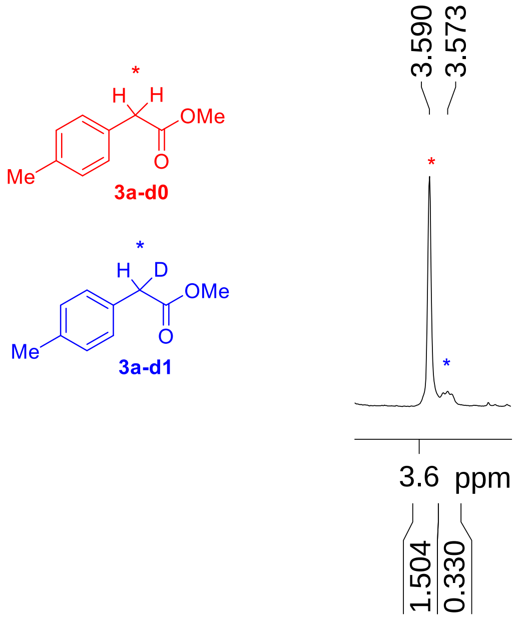
H
H
OMe
Me
O
3a-d0
H
D
OMe
Me
O
3a-d1
3.590
3.573
3.6
ppm
1.504
0.330

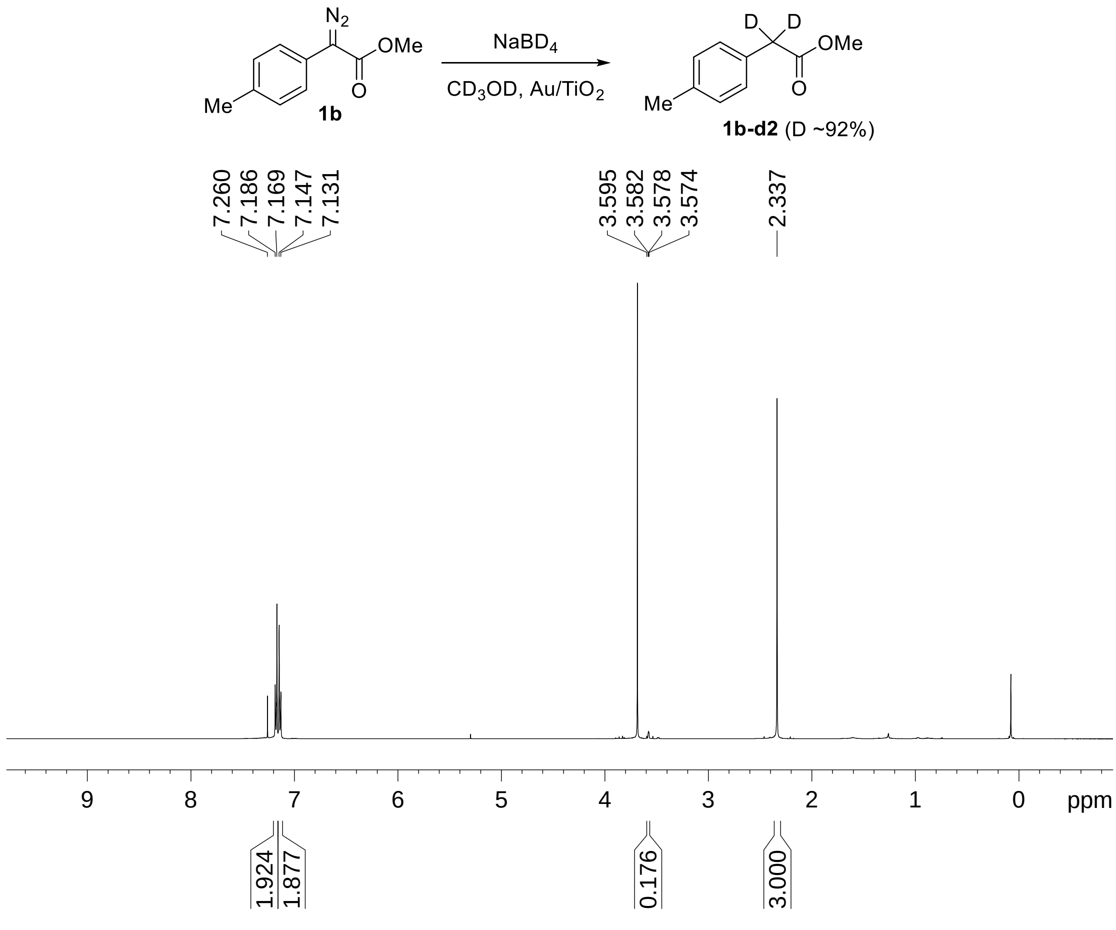

N2
OMe
O
Me
1b
NaBD4
CD3OD, Au/TiO2
D D
OMe
O
Me
1b-d2 (D ~92%)
7.260
7.186
7.169
7.147
7.131
3.595
3.582
3.578
3.574
2.337
9
8
7
6
5
4
3
2
1
0
ppm
1.924
1.877
0.176
3.000

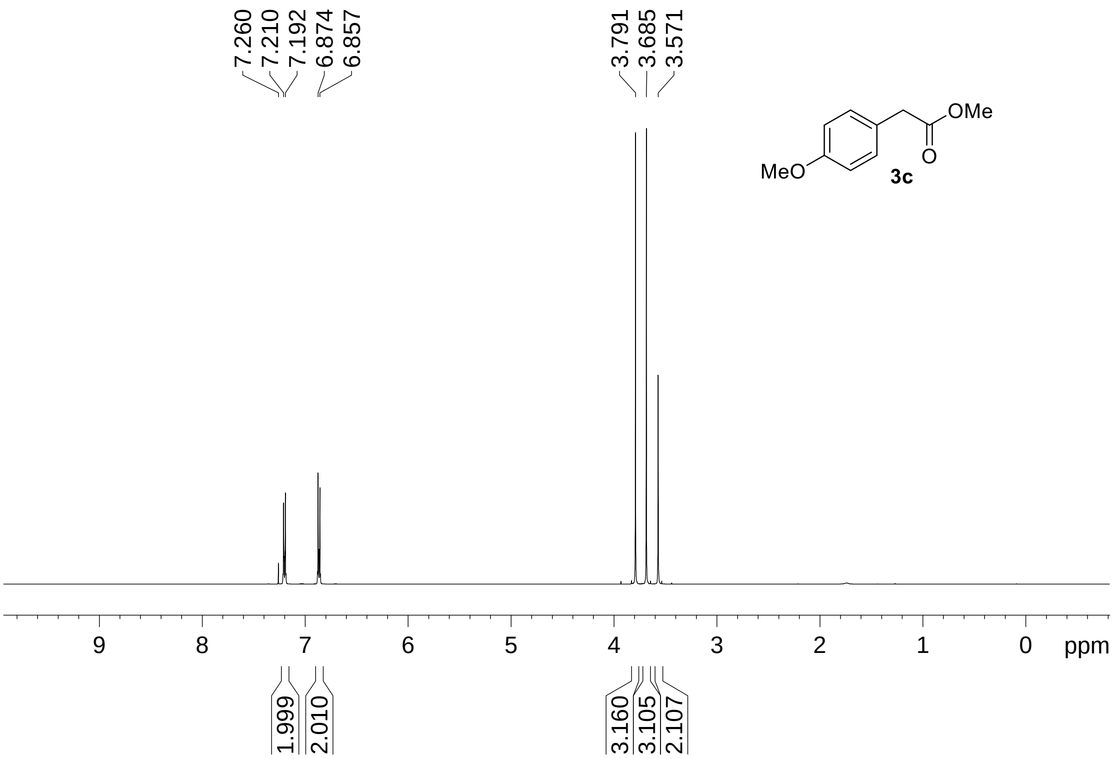
7.260
7.210
7.192
6.874
6.857
3.791
3.685
3.571
OMe
MeO
O
3c
9 8 7 6 5 4 3 2 1 0 ppm
1.999
2.010
3.160
3.105
2.107

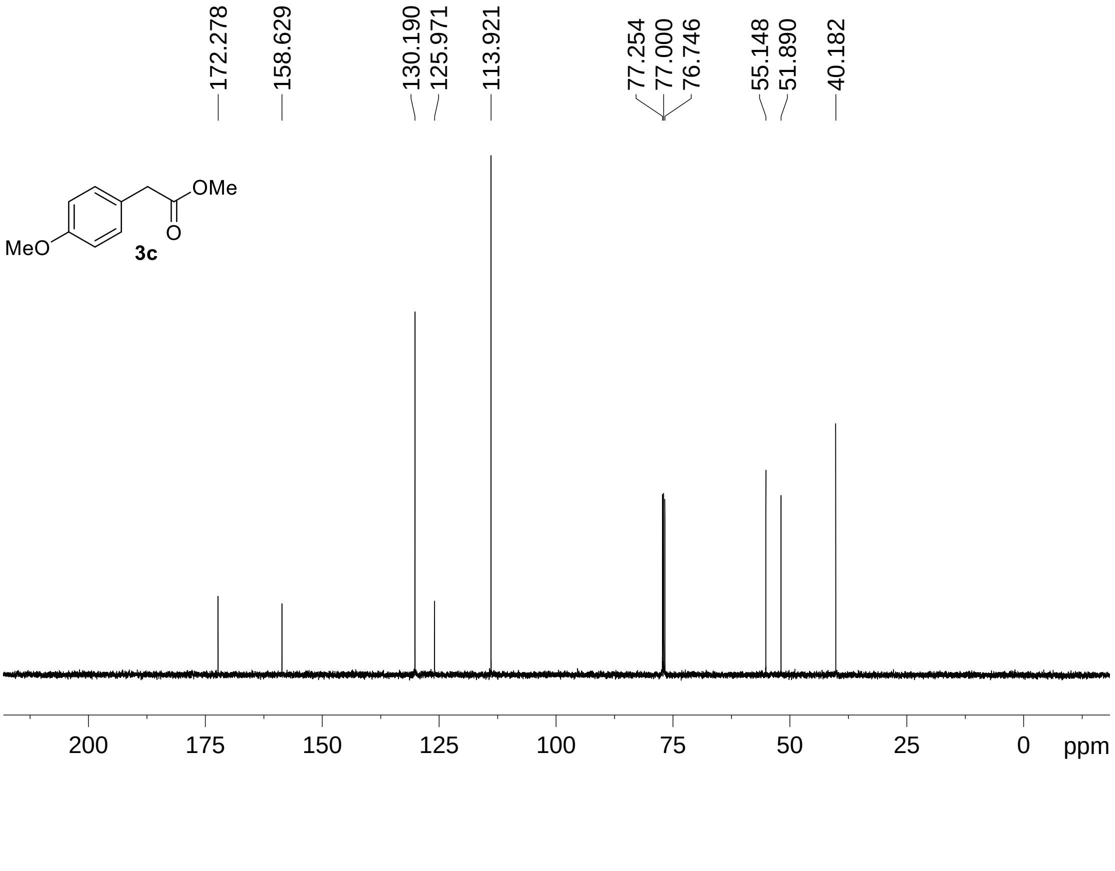
172.278
158.629
130.190
125.971
113.921
77.254
77.000
76.746
55.148
51.890
40.182
OMe
MeO
O
3c
200 175 150 125 100 75 50 25 0 ppm

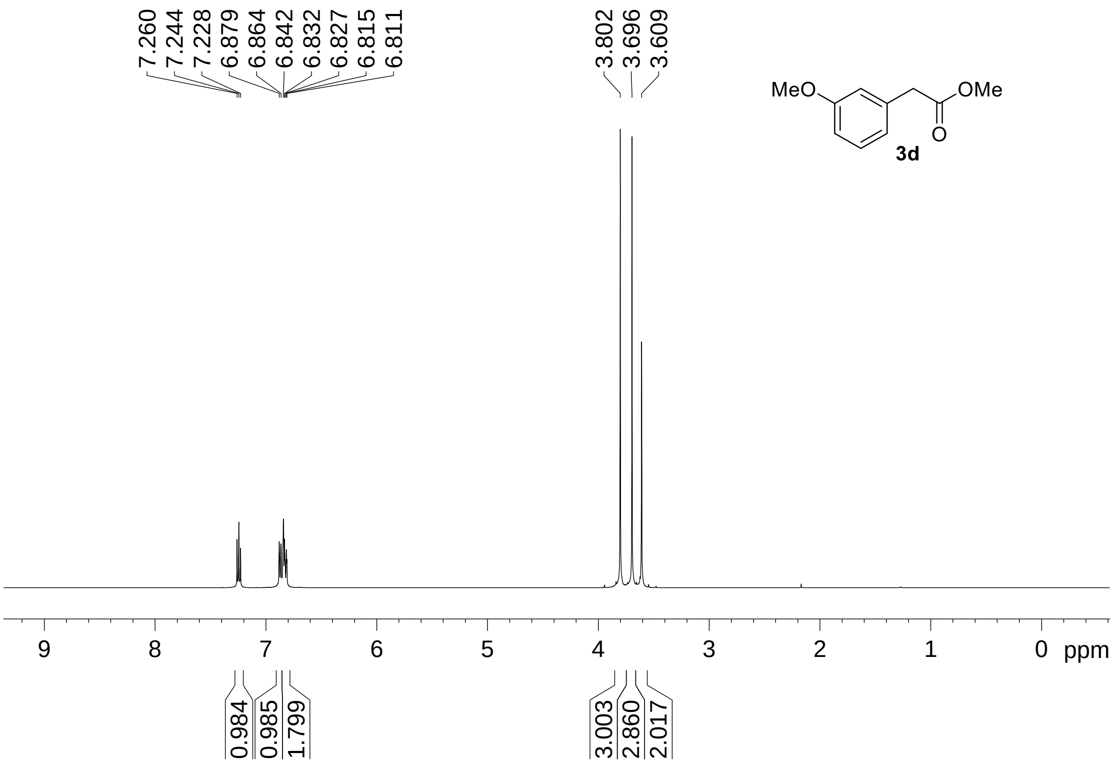
7.260
7.244
7.228
6.879
6.864
6.842
6.832
6.827
6.815
6.811
3.802
3.696
3.609
MeO
OMe
O
3d
9
8
7
6
5
4
3
2
1
0
ppm
0.984
0.985
1.799
3.003
2.860
2.017

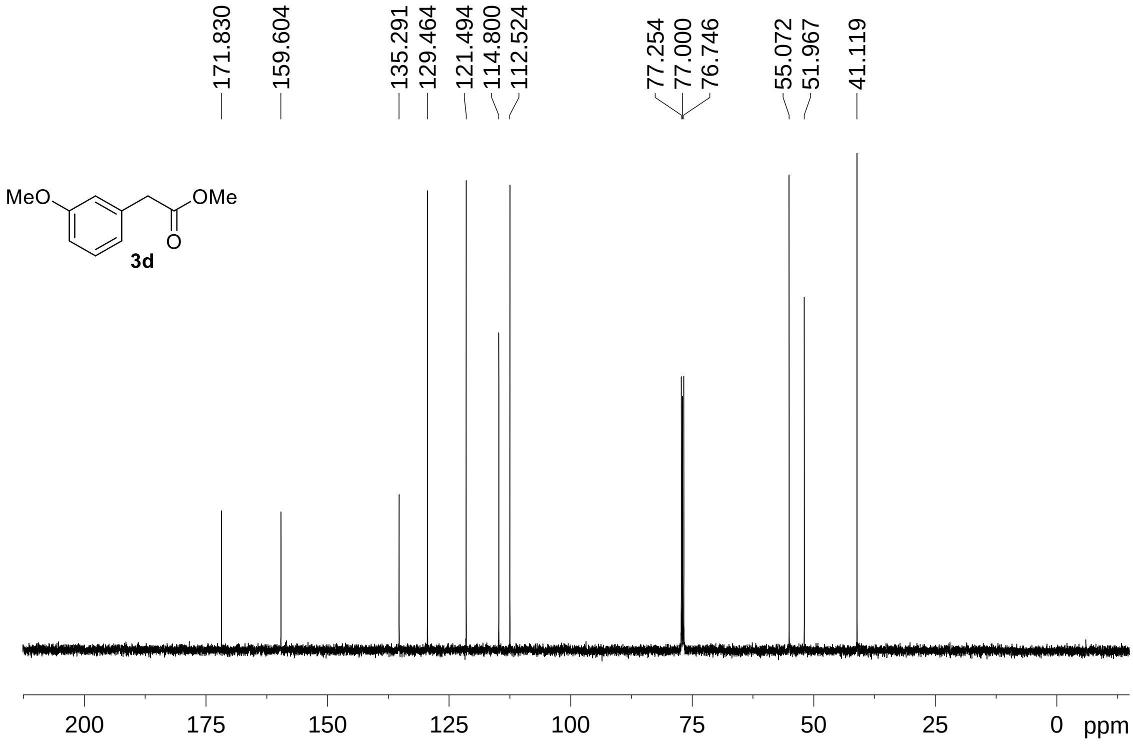
171.830
159.604
135.291
129.464
121.494
114.800
112.524
77.254
77.000
76.746
55.072
51.967
41.119
MeO
OMe
O
3d
200
175
150
125
100
75
50
25
0
ppm

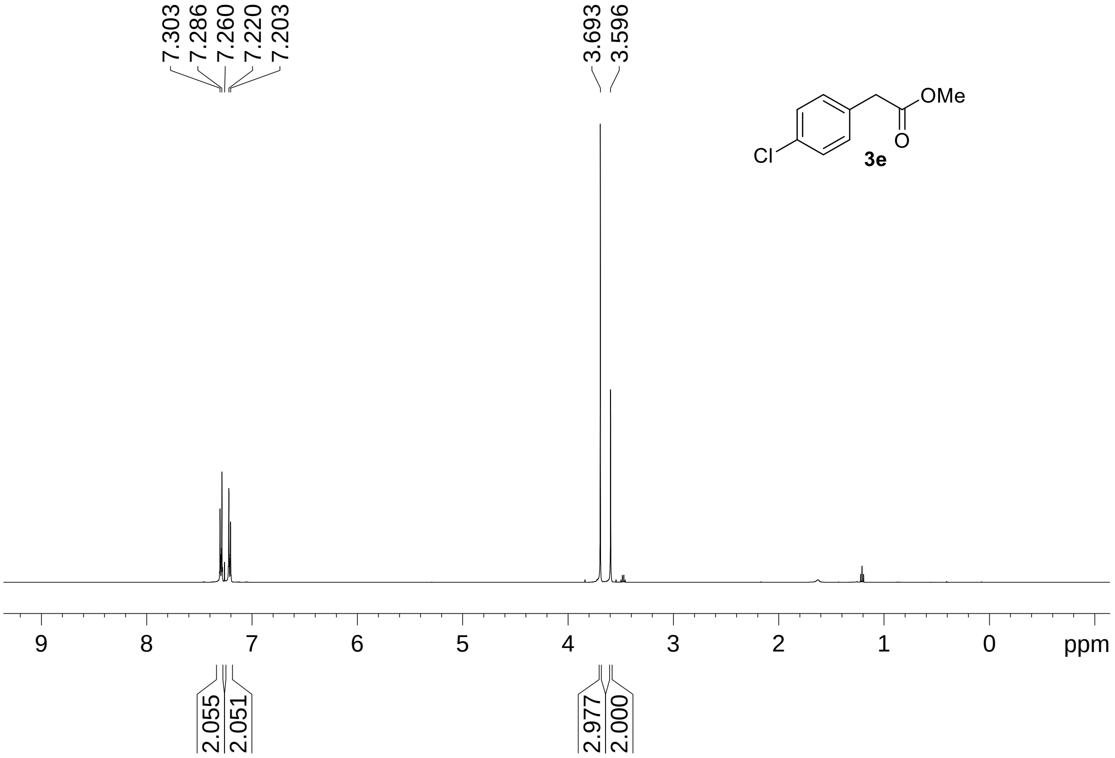
7.303
7.286
7.260
7.220
7.203
3.693
3.596
OMe
O
Cl
3e
9 8 7 6 5 4 3 2 1 0 ppm
2.055
2.051
2.977
2.000

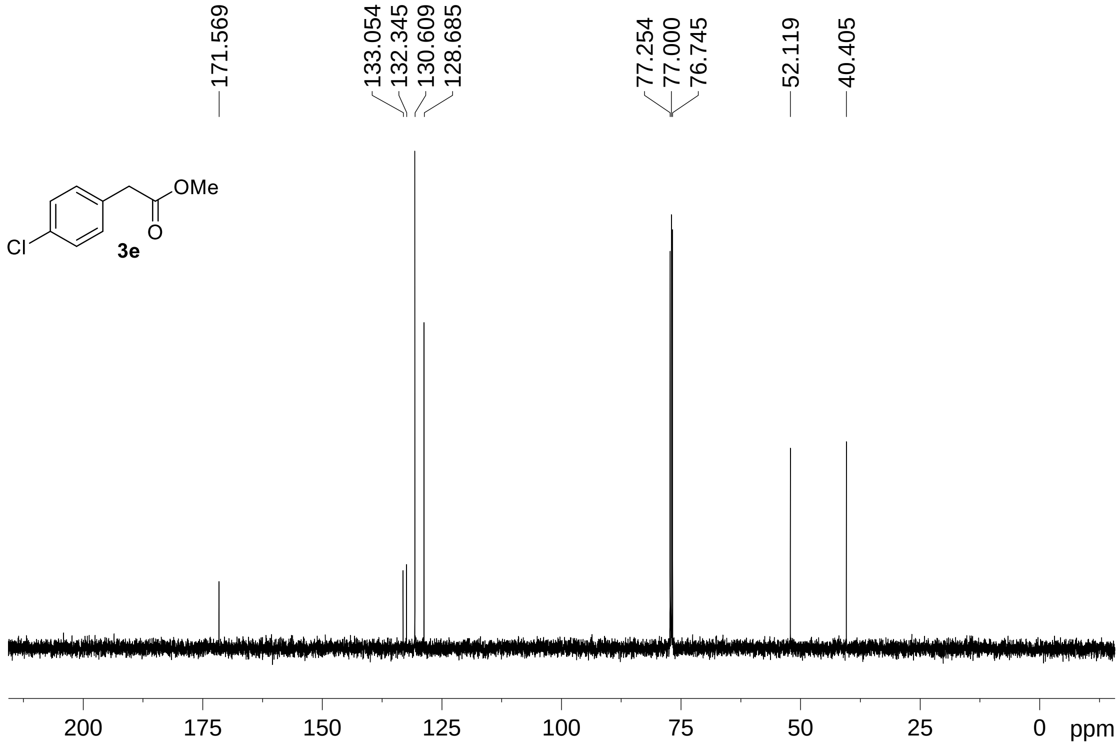
171.569
133.054
132.345
130.609
128.685
77.254
77.000
76.745
52.119
40.405
OMe
O
Cl
3e
200 175 150 125 100 75 50 25 0 ppm

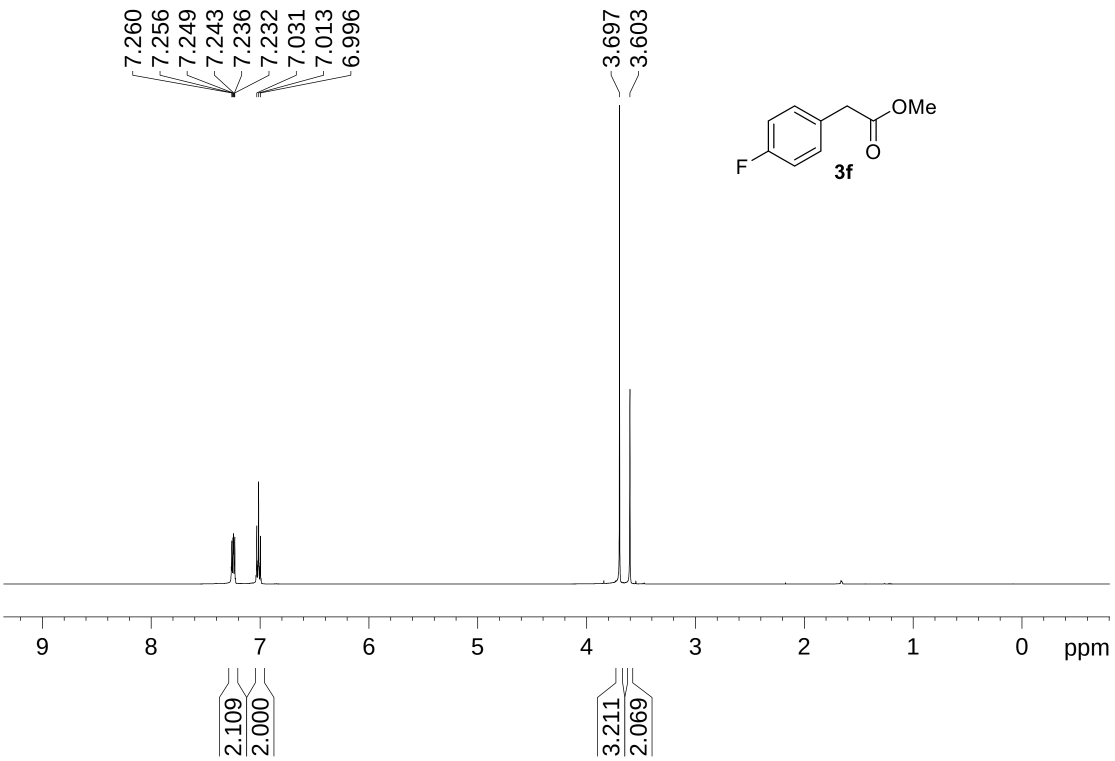
7.260
7.256
7.249
7.243
7.236
7.232
7.031
7.013
6.996
3.697
3.603
OMe
O
F
3f
9
8
7
6
5
4
3
2
1
0
ppm
2.109
2.000
3.211
2.069

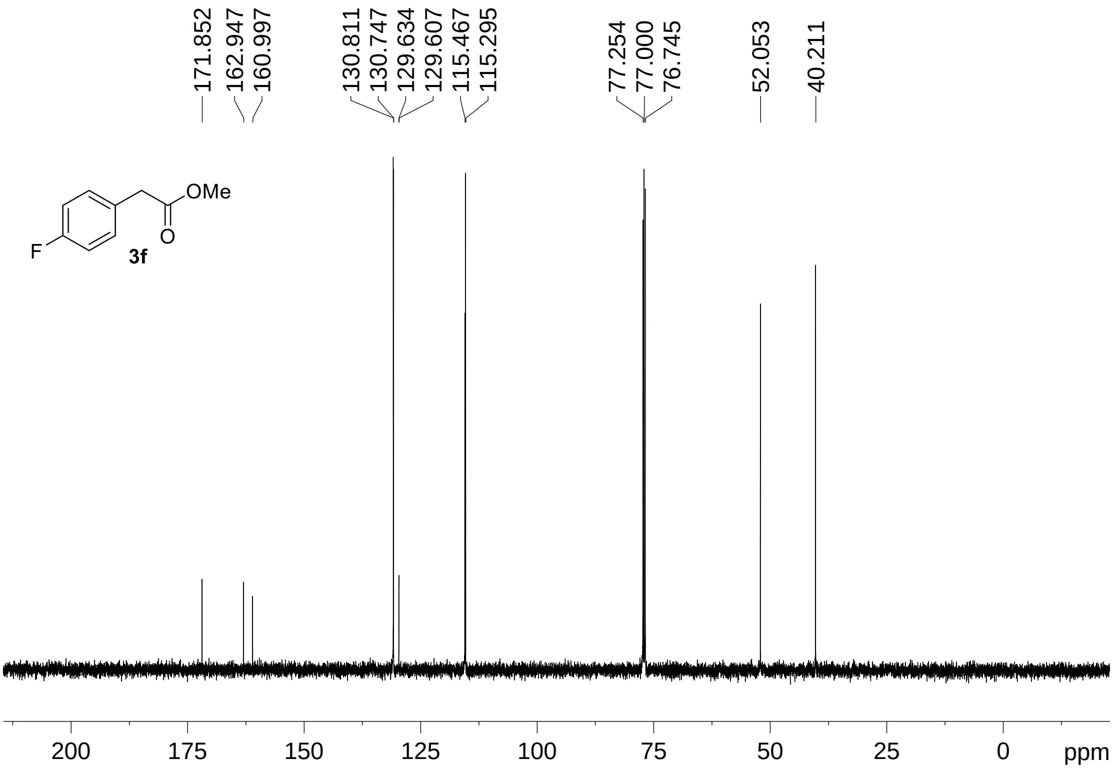
171.852
162.947
160.997
130.811
130.747
129.634
129.607
115.467
115.295
77.254
77.000
76.745
52.053
40.211
OMe
O
F
3f
200
175
150
125
100
75
50
25
0
ppm

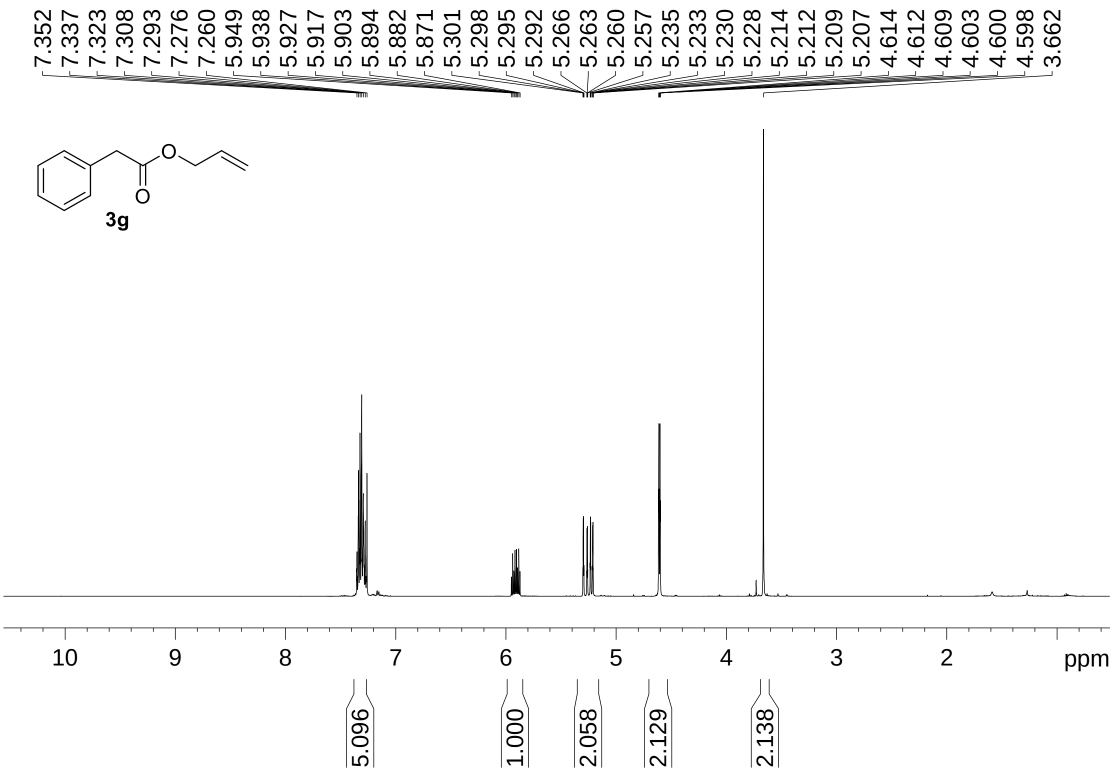
7.352 7.337 7.323 7.308 7.293 7.276 7.260 5.949 5.938 5.927 5.917 5.903 5.894 5.882 5.871 5.301 5.298 5.295 5.292 5.266 5.263 5.260 5.257 5.235 5.233 5.230 5.228 5.214 5.212 5.209 5.207 4.614 4.612 4.609 4.603 4.600 4.598 3.662
3g
10 9 8 7 6 5 4 3 2 ppm
5.096 1.000 2.058 2.129 2.138

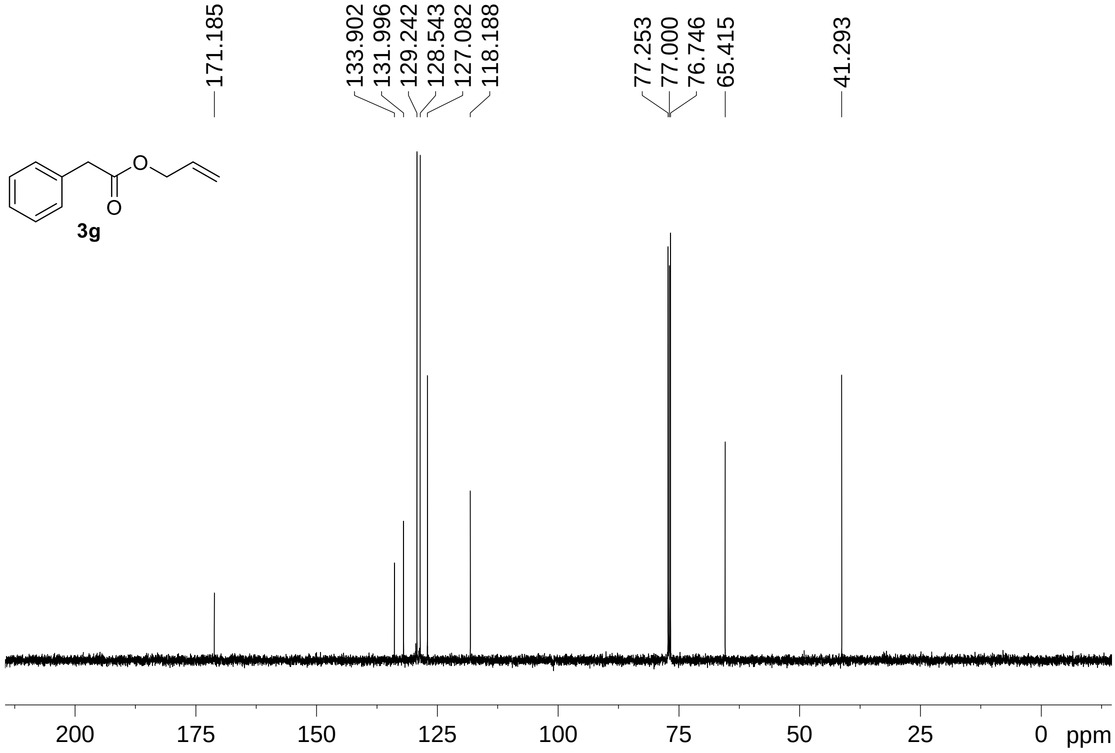
171.185
133.902 131.996 129.242 128.543 127.082 118.188
77.253 77.000 76.746 65.415
41.293
3g
200 175 150 125 100 75 50 25 0 ppm

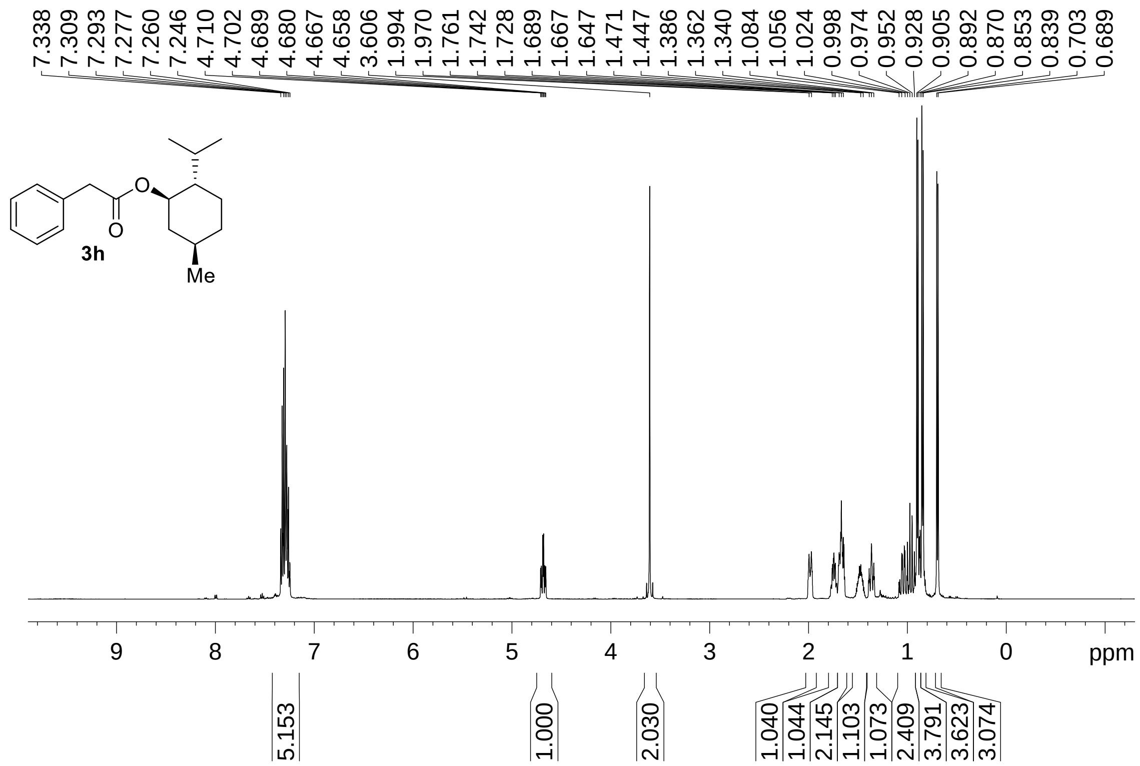
7.338
7.309
7.293
7.277
7.260
7.246
4.710
4.702
4.689
4.680
4.667
4.658
3.606
1.994
1.970
1.761
1.742
1.728
1.689
1.667
1.647
1.471
1.447
1.386
1.362
1.340
1.084
1.056
1.024
0.998
0.974
0.952
0.928
0.905
0.892
0.870
0.853
0.839
0.703
0.689
3h
Me
O
9 8 7 6 5 4 3 2 1 0 ppm
5.153
1.000
2.030
1.040
1.044
2.145
1.103
1.073
2.409
3.791
3.623
3.074

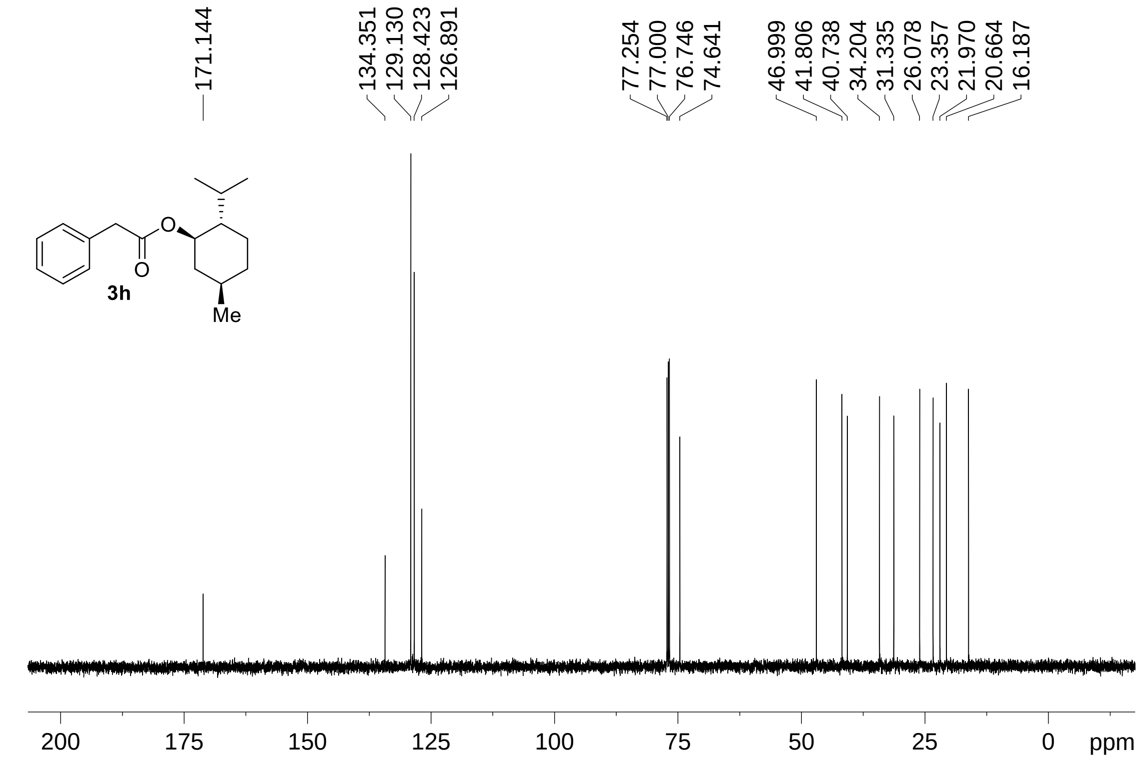
171.144
134.351
129.130
128.423
126.891
77.254
77.000
76.746
74.641
46.999
41.806
40.738
34.204
31.335
26.078
23.357
21.970
20.664
16.187
3h
Me
O
200 175 150 125 100 75 50 25 0 ppm

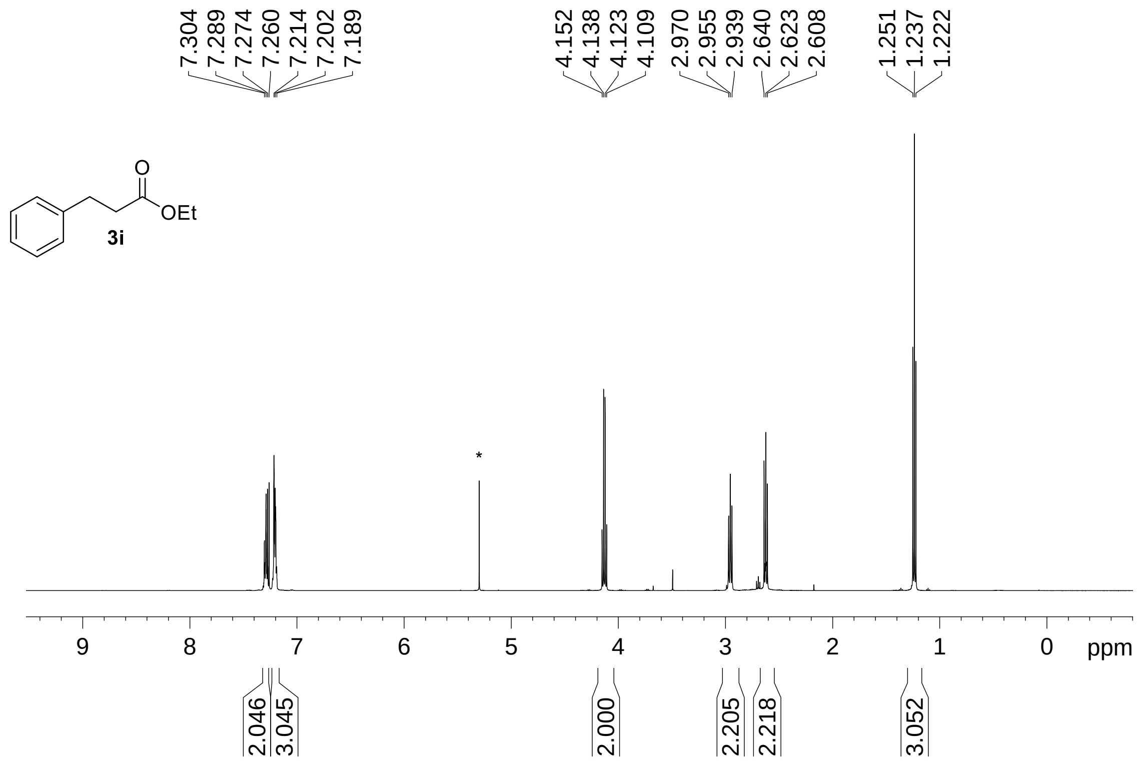
7.304
7.289
7.274
7.260
7.214
7.202
7.189
4.152
4.138
4.123
4.109
2.970
2.955
2.939
2.640
2.623
2.608
1.251
1.237
1.222
O
OEt
3i
*
9
8
7
6
5
4
3
2
1
0
ppm
2.046
3.045
2.000
2.205
2.218
3.052

*DCM

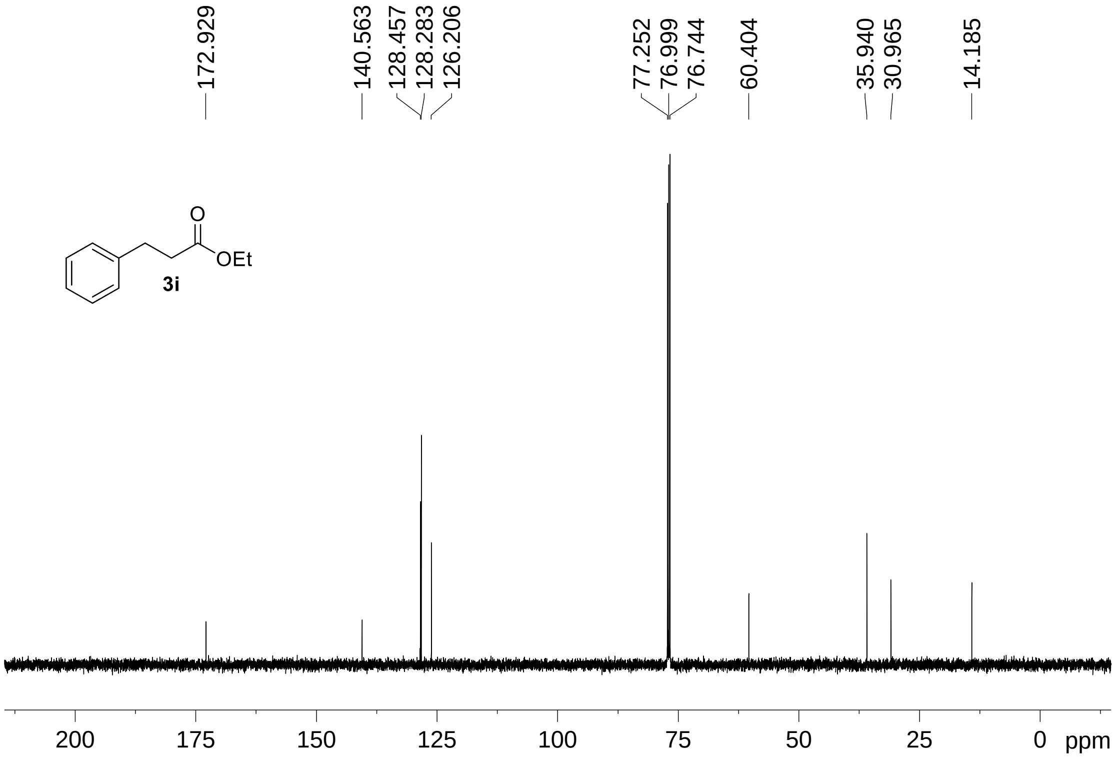
172.929
140.563
128.457
128.283
126.206
77.252
76.999
76.744
60.404
35.940
30.965
14.185
O
OEt
3i
200
175
150
125
100
75
50
25
0
ppm

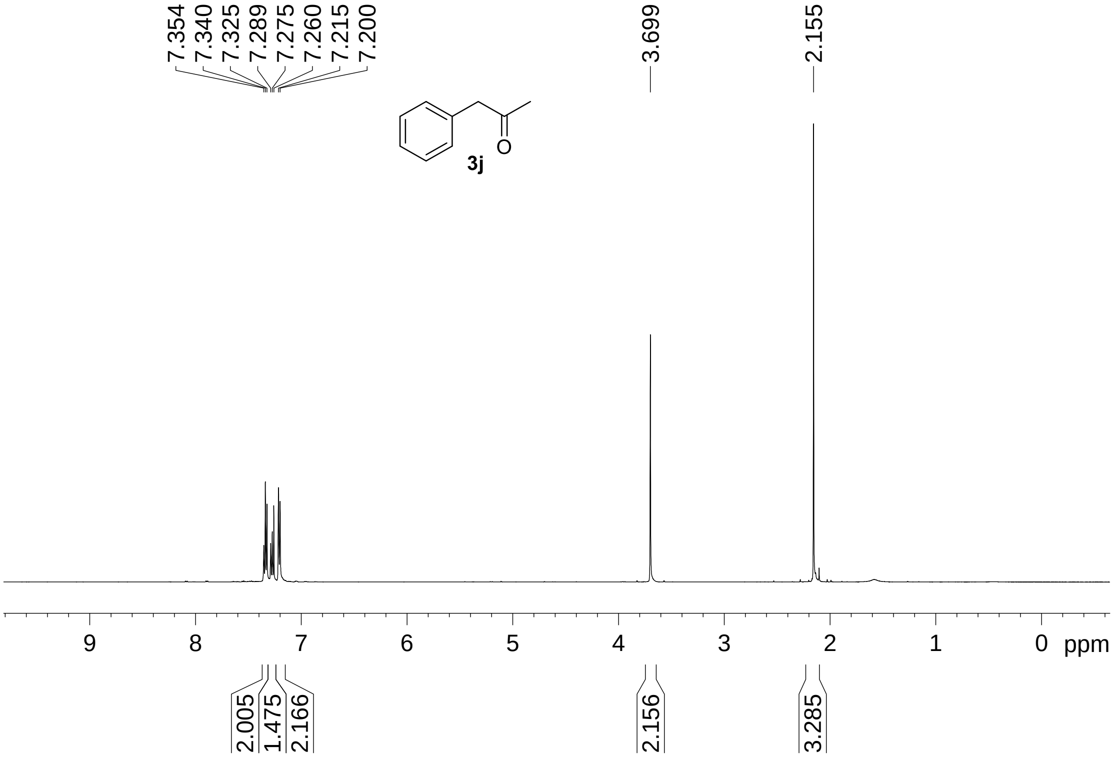
7.354
7.340
7.325
7.289
7.275
7.260
7.215
7.200
3.699
2.155
O
3j
9
8
7
6
5
4
3
2
1
0
ppm
2.005
1.475
2.166
2.156
3.285

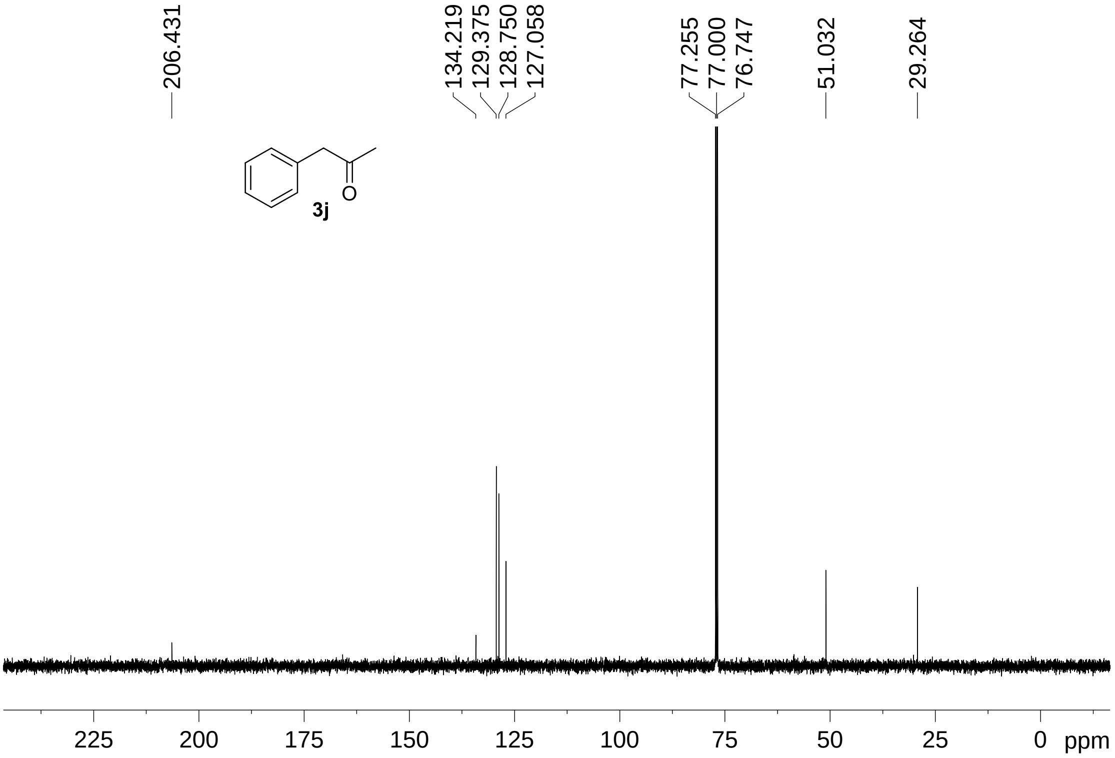
206.431
134.219
129.375
128.750
127.058
77.255
77.000
76.747
51.032
29.264
O
3j
225
200
175
150
125
100
75
50
25
0
ppm

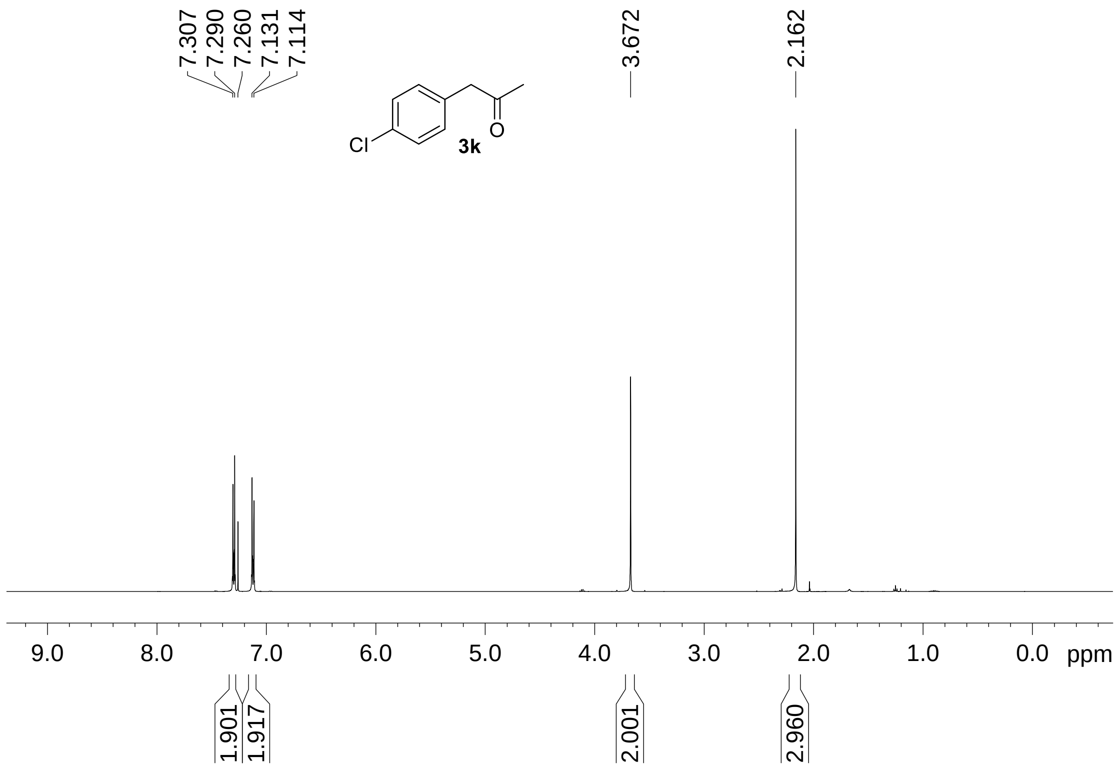
7.307
7.290
7.260
7.131
7.114
3.672
2.162
Cl
O
3k
9.0 8.0 7.0 6.0 5.0 4.0 3.0 2.0 1.0 0.0 ppm
1.901
1.917
2.001
2.960

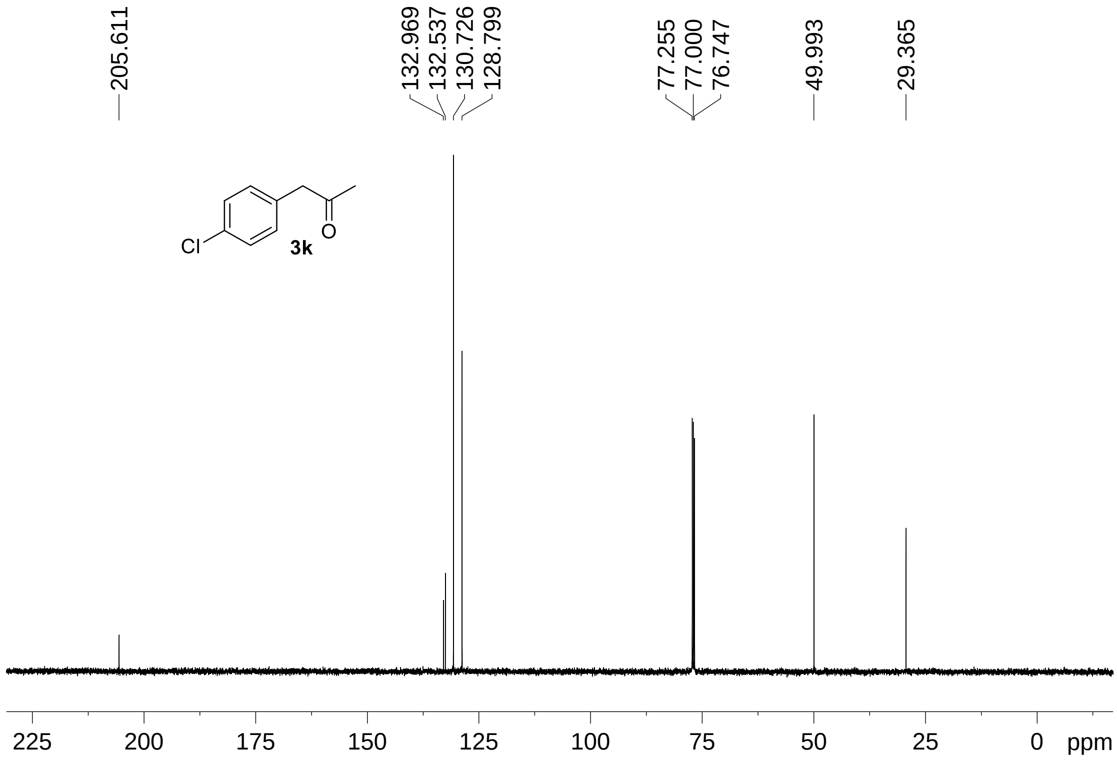
205.611
132.969
132.537
130.726
128.799
77.255
77.000
76.747
49.993
29.365
Cl
O
3k
225 200 175 150 125 100 75 50 25 0 ppm

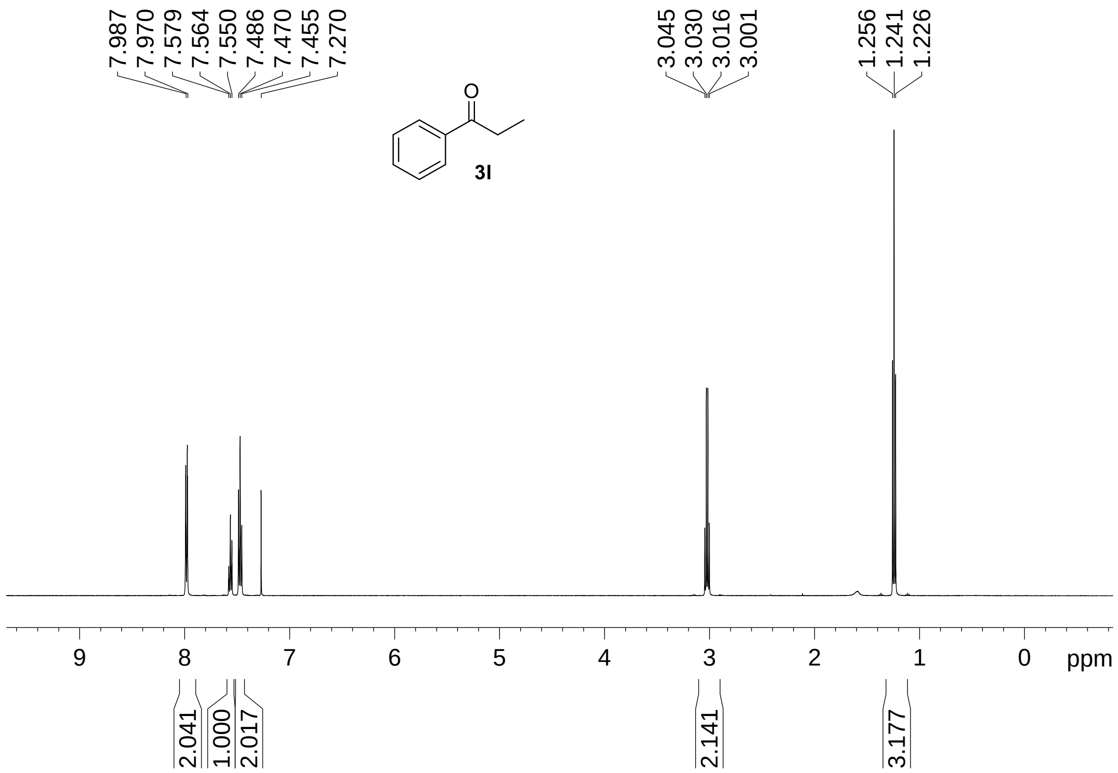
7.987
7.970
7.579
7.564
7.550
7.486
7.470
7.455
7.270
3.045
3.030
3.016
3.001
1.256
1.241
1.226
O
3l
9
8
7
6
5
4
3
2
1
0
ppm
2.041
1.000
2.017
2.141
3.177

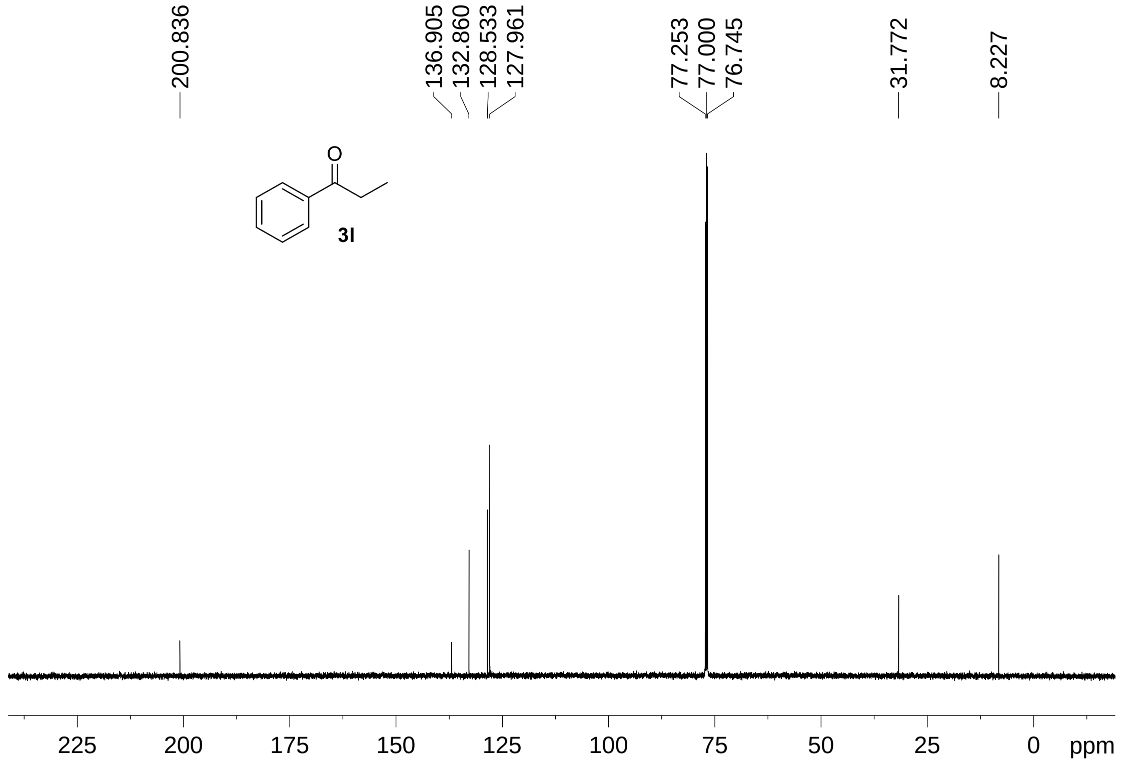
200.836
136.905
132.860
128.533
127.961
77.253
77.000
76.745
31.772
8.227
O
3l
225
200
175
150
125
100
75
50
25
0
ppm

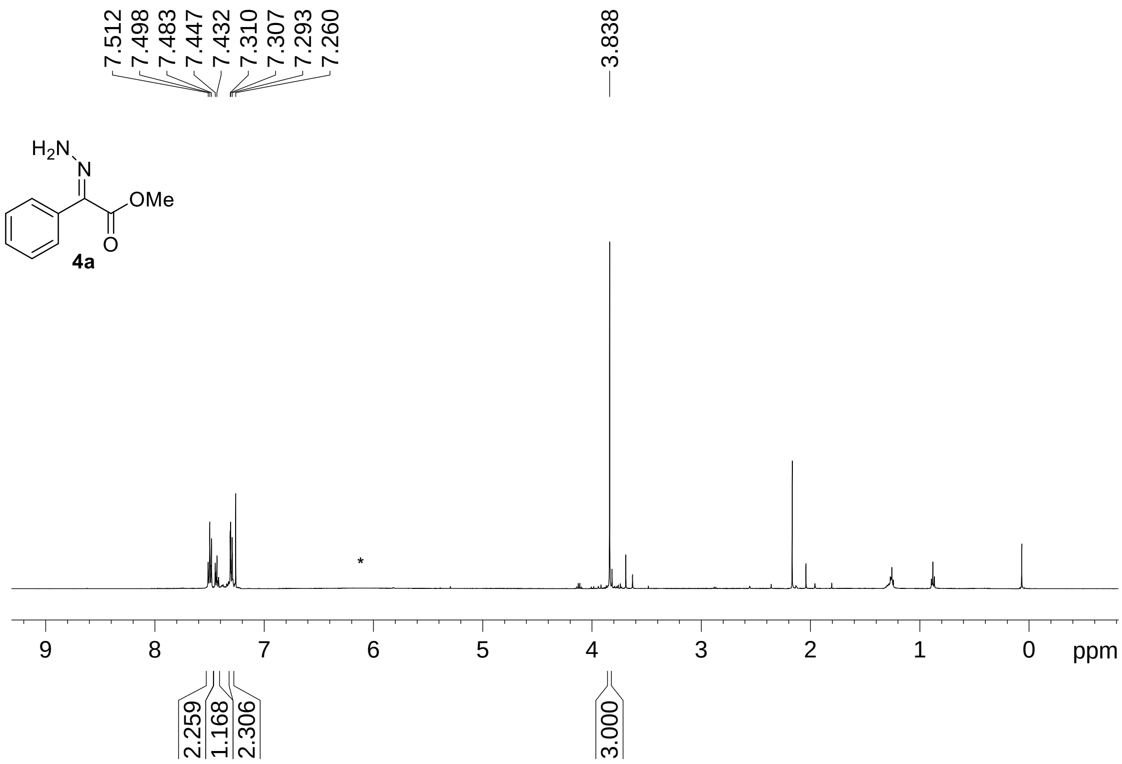

*The peak of NH_2 group is a quite broad singlet centered around 6.2 ppm.

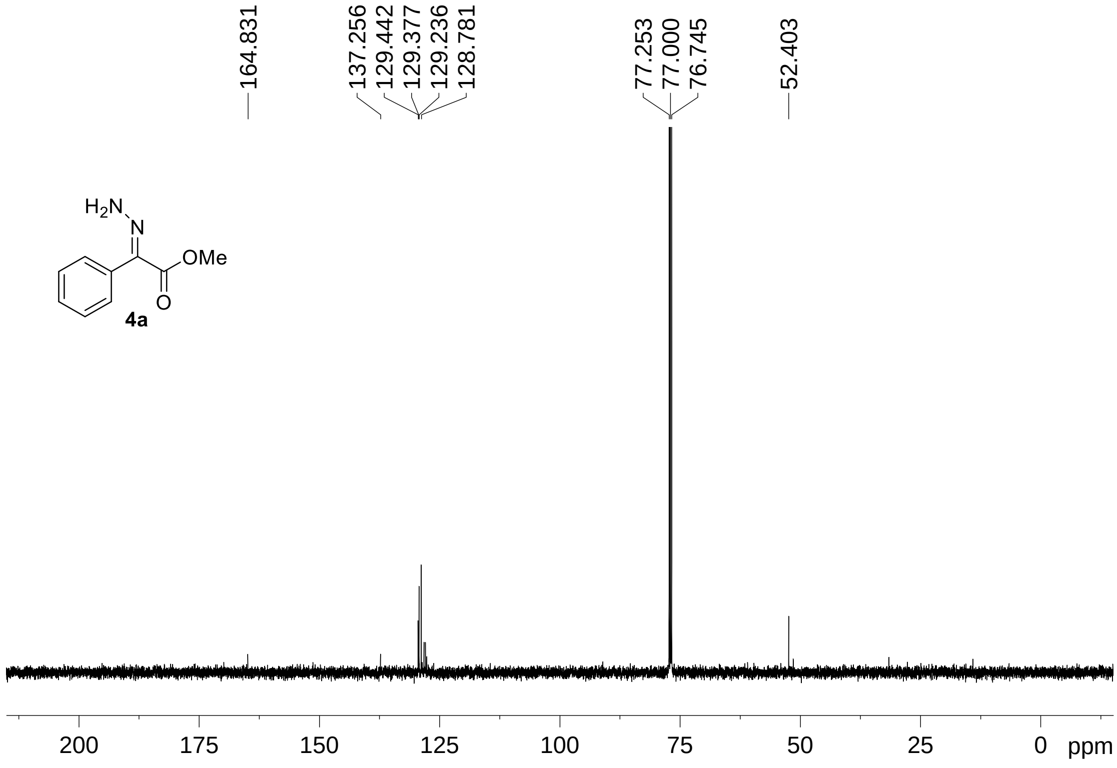

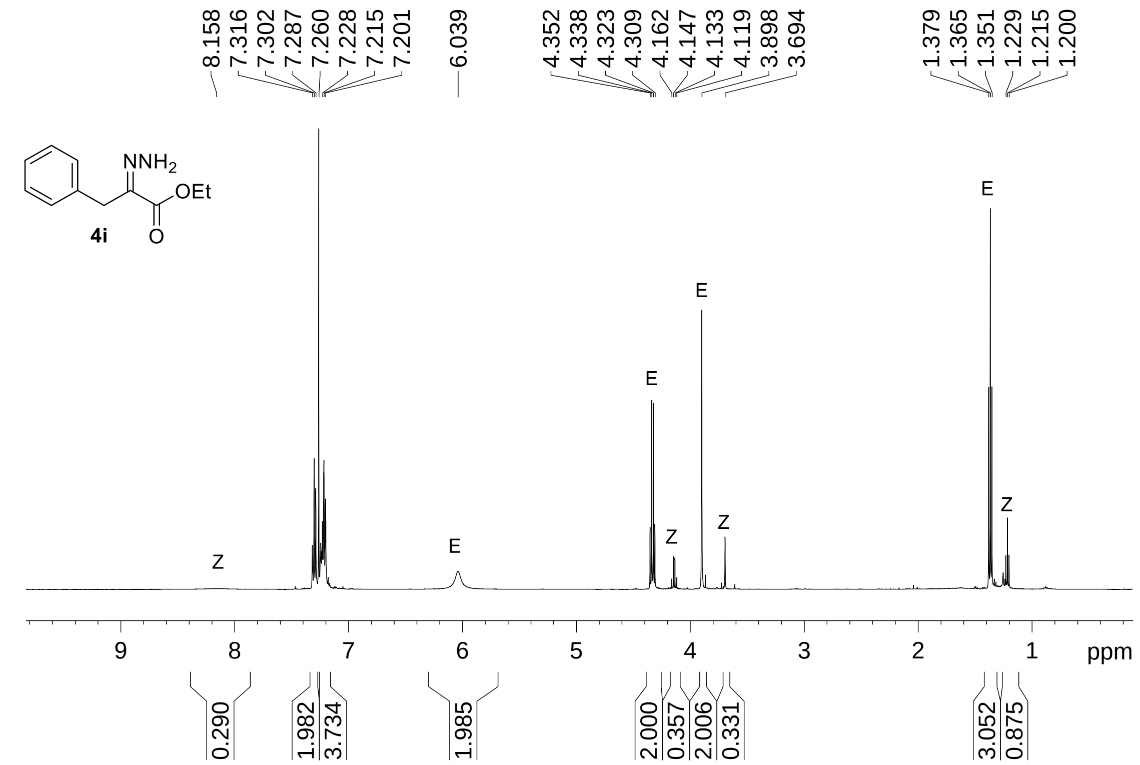

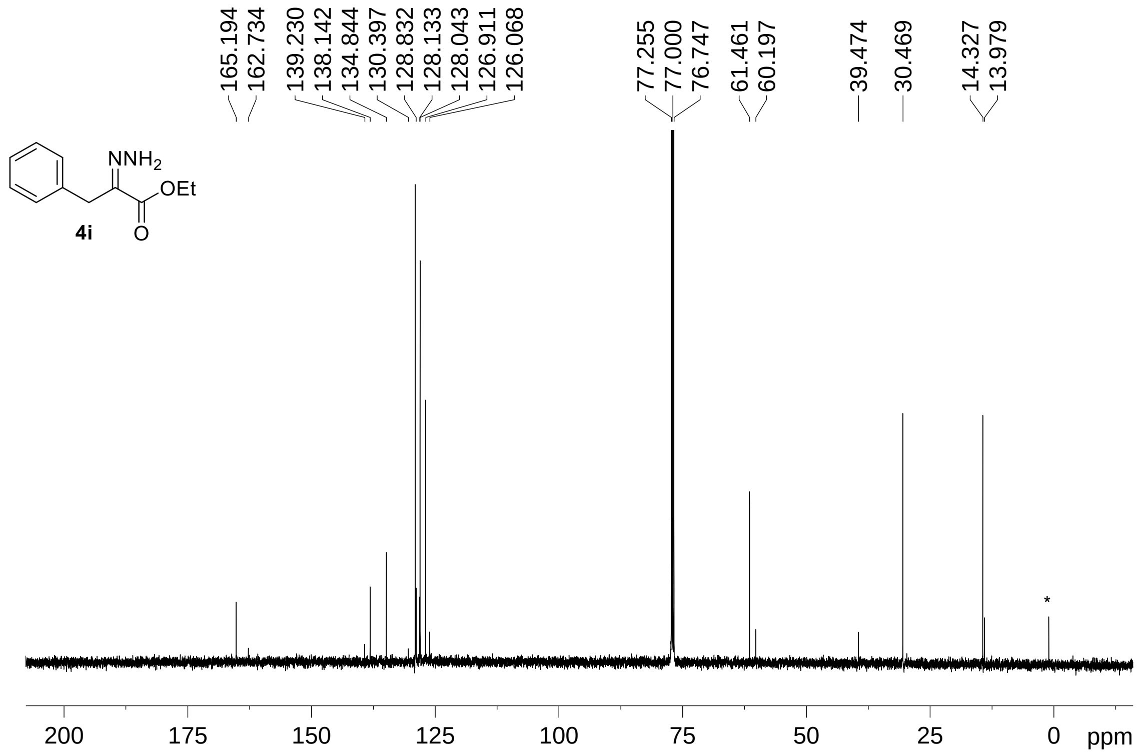

*Silicon grease

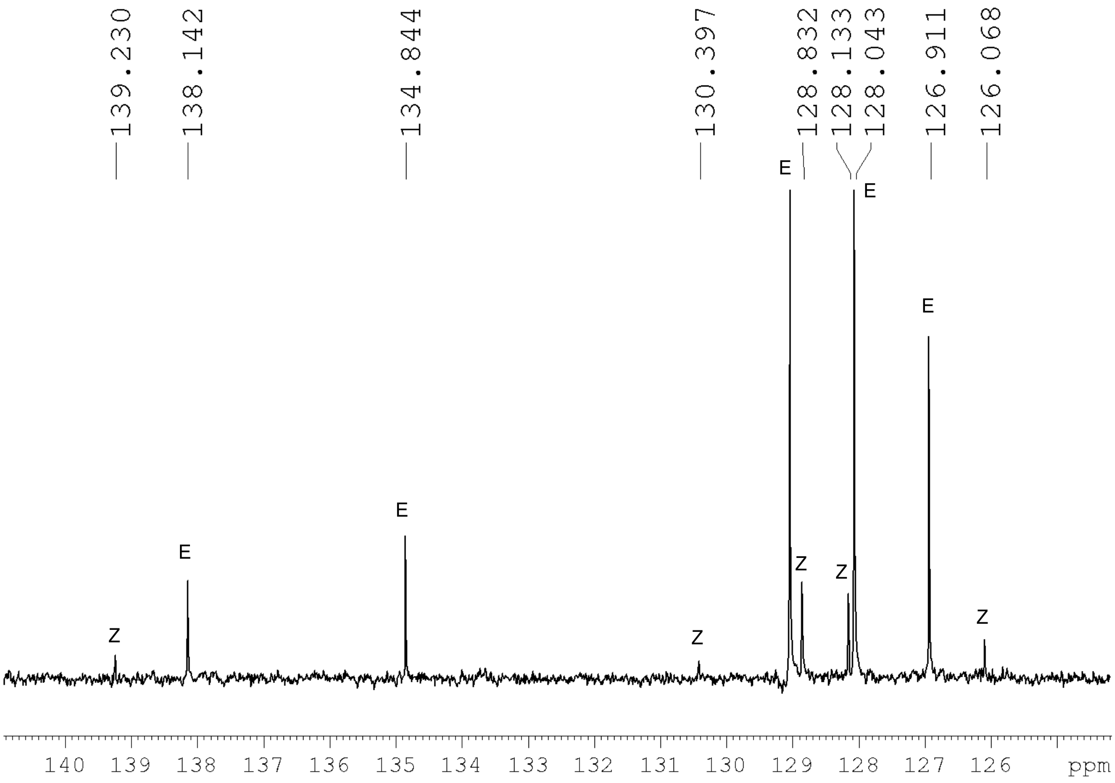

139.230
138.142
134.844
130.397
128.832
128.133
128.043
126.911
126.068
Z
E
E
Z
E
Z
Z
E
E
Z
140 139 138 137 136 135 134 133 132 131 130 129 128 127 126 ppm

Crude ^{11}B NMR spectra showing the boron-bearing byproducts arising from NH_3BH_3 and $NaBH_4$ in the reduction of α-diazocarbonyl compounds in CD_3OD under the Au/TiO_2-catalyzed reaction conditions

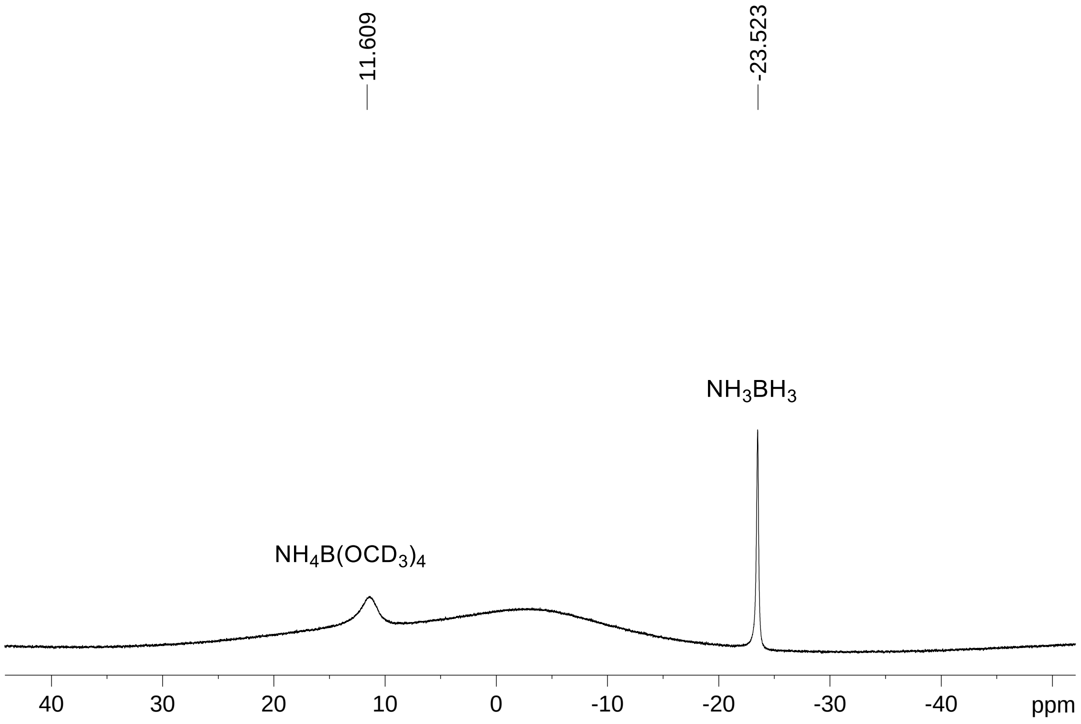

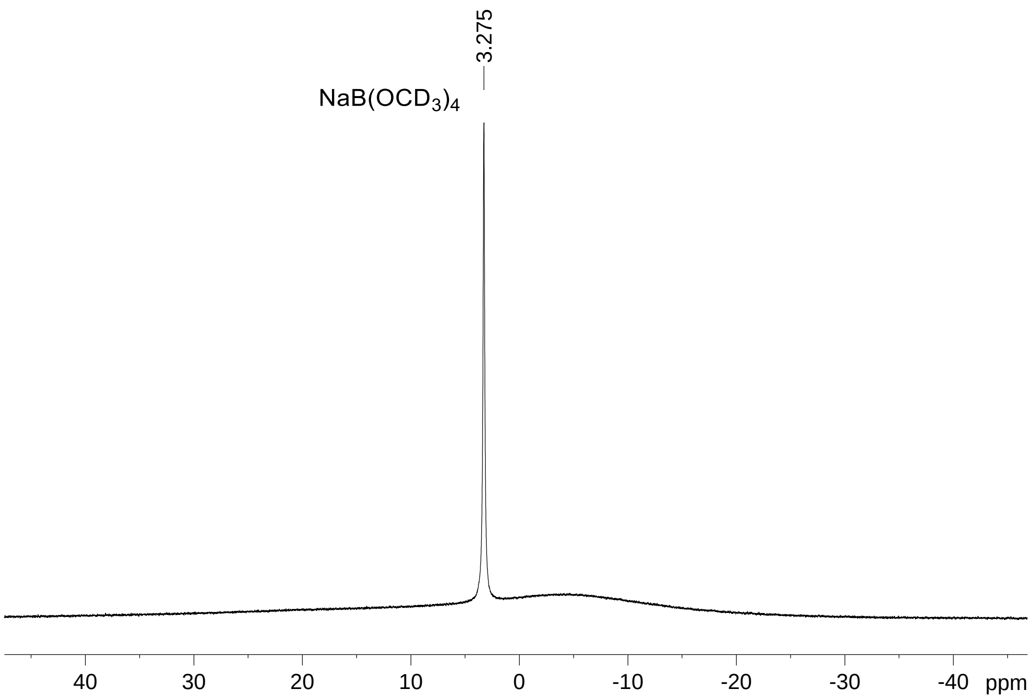

Supplement: Supplementary file 1 [file nanomaterials-11-00248-s001.pdf]
